# Perturbation of B Cell Gene Expression Persists in HIV-Infected Children Despite Effective Antiretroviral Therapy and Predicts H1N1 Response

**DOI:** 10.3389/fimmu.2017.01083

**Published:** 2017-09-11

**Authors:** Nicola Cotugno, Lesley De Armas, Suresh Pallikkuth, Stefano Rinaldi, Biju Issac, Alberto Cagigi, Paolo Rossi, Paolo Palma, Savita Pahwa

**Affiliations:** ^1^Research Unit in Congenital and Perinatal Infection, Immune and Infectious Diseases Division, Academic Department of Pediatrics, Bambino Gesù Children’s Hospital, Rome, Italy; ^2^Miami Center for AIDS Research, Department of Microbiology and Immunology, Miller School of Medicine, University of Miami, Miami, FL, United States; ^3^Sylvester Cancer Center, Department of Biostatistics and Bioinformatics, Miller School of Medicine, University of Miami, Miami, FL, United States; ^4^Vaccine Research Center, National Institute of Allergy and Infectious Diseases (NIAID), National Institutes of Health (NIH), Bethesda, MD, United States; ^5^Academic Department of Pediatrics (DPUO), Bambino Gesù Children’s Hospital-University of Rome Tor Vergata, Rome, Italy

**Keywords:** vaccinomics, systems biology, B cells, pediatric HIV, transcriptomics, H1N1, B cell receptor, influenza vaccination

## Abstract

Despite effective antiretroviral therapy (ART), HIV-infected individuals with apparently similar clinical and immunological characteristics can vary in responsiveness to vaccinations. However, molecular mechanisms responsible for such impairment, as well as biomarkers able to predict vaccine responsiveness in HIV-infected children, remain unknown. Following the hypothesis that a B cell qualitative impairment persists in HIV-infected children (HIV) despite effective ART and phenotypic B cell immune reconstitution, the aim of the current study was to investigate B cell gene expression of HIV compared to age-matched healthy controls (HCs) and to determine whether distinct gene expression patterns could predict the ability to respond to influenza vaccine. To do so, we analyzed prevaccination transcriptional levels of a 96-gene panel in equal numbers of sort-purified B cell subsets (SPBS) isolated from peripheral blood mononuclear cells using multiplexed RT-PCR. Immune responses to H1N1 antigen were determined by hemaglutination inhibition and memory B cell ELISpot assays following trivalent-inactivated influenza vaccination (TIV) for all study participants. Although there were no differences in terms of cell frequencies of SPBS between HIV and HC, the groups were distinguishable based upon gene expression analyses. Indeed, a 28-gene signature, characterized by higher expression of genes involved in the inflammatory response and immune activation was observed in activated memory B cells (CD27^+^CD21^−^) from HIV when compared to HC despite long-term viral control (>24 months). Further analysis, taking into account H1N1 responses after TIV in HIV participants, revealed that a 25-gene signature in resting memory (RM) B cells (CD27^+^CD21^+^) was able to distinguish vaccine responders from non-responders (NR). In fact, prevaccination RM B cells of responders showed a higher expression of gene sets involved in B cell adaptive immune responses (*APRIL, BTK, BLIMP1*) and BCR signaling (*MTOR, FYN, CD86*) when compared to NR. Overall, these data suggest that a perturbation at a transcriptional level in the B cell compartment persists despite stable virus control achieved through ART in HIV-infected children. Additionally, the present study demonstrates the potential utility of transcriptional evaluation of RM B cells before vaccination for identifying predictive correlates of vaccine responses in this population.

## Introduction

HIV-infected patients have a lower ability to induce and maintain an effective response to routine vaccinations due to the depletion of central memory CD4 T cells, particularly T follicular helper cells, and perturbation of the B cell compartment with reduced resting memory (RM) B cells (1–4). Antiretroviral therapy (ART) can restore the quantitative loss of RM B cells in HIV-infected children (5, 6). However, a suboptimal antibody response against infection and vaccination may persist, suggesting a qualitative impairment of B cells. Indeed, a sizeable proportion of HIV-infected children require booster immunizations to provide adequate protection usually achieved by routine vaccinations in healthy children (7–9). Additionally, children with apparently similar clinical and immunological characteristics can vary in adequacy of responsiveness to infection and/or vaccination bringing into question host factors that are critical for mounting an immune response (10, 11). The molecular correlates governing effective and long lasting immune responses are still unknown (4, 12–14). In recent years, systems biology and vaccinomics approaches have attempted to dissect vaccine-induced responses in humans (15–19). For influenza, gene expression and robustness of response have been found to differ upon vaccination with trivalent-inactivated influenza vaccination (TIV) as compared to live attenuated influenza vaccine (20). In addition, advanced “omics” and systems biology approaches have led to increased knowledge regarding molecular mechanisms underlying adaptive immune responses to different types of vaccines (21, 22). In most instances however, these data have been derived from RNA extracted from whole blood or from the heterogeneous pool of peripheral blood mononuclear cells (PBMCs) of healthy volunteers (18, 23), thereby limiting interpretation due to dilution of gene transcripts derived from individual cell subsets or single cells which may be crucial for adaptive immune responses. To mitigate this drawback, analysis of purified cell subsets of interest is preferred, especially in the context of diseases that alter the distribution of specific cell subsets such as HIV infection (24, 25).

In the present study, we have applied basic principles of vaccinomics and systems biology, with the aim to dissect gene expression differences evident before vaccine administration between HIV-infected children under ART with stable virus control and their age matched healthy peers. Our analysis of B cell gene expression among HIV-infected children differentially responding to H1N1 revealed biologically meaningful predictive signatures of response to vaccination.

## Materials and Methods

### Study Subjects and Design

Twenty-three ART-treated HIV-1 vertically infected patients (HIV) and 10 healthy age-matched controls [healthy controls (HCs)] were enrolled at Bambino Gesù Children’s hospital. Participant characteristics are shown in Table 1. Written informed consent was obtained from all subjects or parents/guardians of all minors for participation in a prospective, open label influenza vaccine study (Figure S1A in Supplementary Material). Bambino Gesù Children’s hospital ethics committee approved the study. Participants were immunized with a single dose of Inactivated Influenza Vaccine Trivalent Types A and B (Split Virion) VAXIGRIP^®^ (sanofi pasteur). The strains for the 2012–2013 season were A/California/7/2009 (H1N1) pdm09-like strain (abbreviated as H1N1), A/Victoria/361/2011 (H3N2)-like strain (abbreviated as H3N2), and B/Wisconsin/1/2010-like strain (abbreviated as B). Study design is outlined in Figure S1A in Supplementary Material. PBMCs, sera, and plasma were collected pre (T0) and 21 days postvaccination (T1) as previously described (26, 27). Among HIV, only patients with good adherence to ART and with history of long-term viral control (at least 24 months) were considered eligible for the study. No significant differences for ART type nor treatment duration were found between HIV Responders and HIV non-responders (NR, Table 1).

**Table 1:** Characteristics of study population.

Baseline characteristics	HIV NR	HIV R	HC
Age (years), mean (SEM)	15.16 (2.1)	13.72 (2.3)	14.3 (3.3)
*n* (female)	12 (7)	11 (5)	10 (5)
%CD4^+^ T cells, mean (SEM)	37.97 (4.9)	32.49 (6.0)	29.79 (6.2)
HIV RNA <50 cp/mL, *n*	11	10	N/A
IgG (mg/dL) (mean)	1,387.4	1,356	1,054.7
IgM (mg/dL) (mean)	135.1	118.9	106.8
IgA (mg/dL) (mean)	210.7	225.1	150
CDC (A/B/C) (1/2/3)	(3/4/5) (3/4/5)	(2/5/4) (4/3/4)	N/A
Lymphocytes/mm^3^, mean (SEM)	2,494 (278.9)	3,109 (363.1)	3,063 (427.8)
WBC (10^3^/μL), mean (SEM)	7.6 (1.5)	7.3 (0.7)	7.9 (0.5)
ART regimen (2 NRTI + PI/2 NRTI + nNRTI/2 NRTI + ii)	(5/5/2)	(5/4/2)	N/A

*CDC, Center for Disease Control classification of AIDS; WBC, white blood cells; ART, antiretroviral treatment; NRTI, nucleoside and nucleotide analog reverse transcriptase inhibitors; PI, protease inhibitors; nNRTI, non-nucleoside analog reverse transcriptase inhibitors; ii, integrase inhibitors*.

### Hemagglutination Inhibition (HAI) Assay

The HAI assay was performed and analyzed as previously described (28) (http://www.gmp-compliance.org/guidemgr/files/021496EN.PDF). The HAI antibody titers were expressed as the reciprocal of the highest serum dilution at which hemagglutination was prevented. Study participants were classified as vaccine responders (R) and vaccine NR according to the criteria established by Food and Drug Administration Guidance for Industry (fda.gov). R were characterized by HAI titer to H1N1 at T1 of ≥1:40 *and* ≥4-fold increase compared to baseline.

### ELISpot

Peripheral blood mononuclear cells collected at T0 and T1 from HIV and HC were thawed and polyclonally activated *in vitro* in complete RPMI medium (Invitrogen) supplemented with 2.5 µg/mL CpG type B (Hycult biotech), 20 ng/mL IL-4 (Peprotech), and 20 ng/mL IL-21 (ProSpec). Cells were harvested after 5 days of culture at 37°C. ELISpot 96-well filtration plates (Millipore) were coated with purified H1N1 inactivated virus particles and subsequently loaded with 2 × 10^5^ cells/well. Plates were then processed as previously described (2). Response to H1N1 Ag was determined using the criteria ≥ or <80 spots/10^6^ PBMCs in R and NR, respectively.

### Cell Sorting, RNA Extraction and FACS Analysis

**Figure 1:**
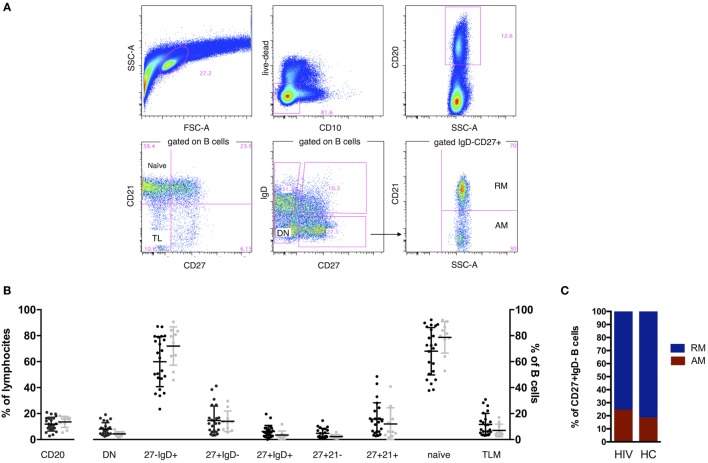
B cell phenotype in HIV and age-matched healthy control (HC). Representative gates **(A)** and comparisons of B cell percentages **(B,C)**. Two tailed Mann–Whitney was used for comparisons. CD20^+^ cells established the B cell population, and expression of IgD, CD27, CD21, and CD10 was used to define total naive (CD27^−^IgD^+^), class switched CD27^+^ memory B cells (CD27^+^IgD^−^), double-negative (DN; CD27^−^IgD^−^), resting memory (RM), tissue-like (TL), activated memory (AM), and naive. FSC, forward scatter; SSC, side scatter. Contingency plot in **(C)** represents frequency of AM and RM in HIV and HC.

Cryopreserved PBMC from T0 and T1 were thawed, stained for the following previously titrated surface antibodies: CD10 (PECy7), CD20 (PE), CD27 (APC), IgD (FITC), CD21 (PECy5), and sorted by FACSAriaII (BD Biosciences). Vivid (Pacific Blue) was used to determine viability of cells. The gating strategy to identify B cell subsets, comprising total B cells (live, singlets, CD20^+^), total naive (IgD^+^CD27^−^), double negative (DN) (IgD^−^CD27^−^), RM (IgD^−^CD27^+^CD21^+^), and activated memory (AM) (IgD^−^CD27^+^CD21^−^) that were gated on the IgD^−^CD27^+^ class switched memory are shown in Figure 1 and Figure S1B in Supplementary Material. IL-21 receptor on B cells has been analyzed as previously described (11). The purity of sorted cell populations was >99%. Five hundred live cells per B cell subset were sorted with the sorting strategy depicted in Figure S1B in Supplementary Material in tubes previously loaded with 9 μL of CellsDirect one-step polymerase chain reaction (PCR) buffer and pooled TaqMan gene expression assays (2× CellsDirect Reaction mix 5 μL, Superscript III + Taq polymerase 0.5 μL, 0.2× TaqMan primer pool 2.5 μL, Resuspension Buffer 1 μL). After sorting, samples were transferred to PCR tubes and reverse transcription and target-specific preamplification was performed on a C1000 Thermal Cycler (BioRad) with the following scheme (50°C for 20 min, 95°C for 2 min, 95°C for 15 s, 60°C for 4 min, last two steps repeated for 18 cycles). Resulting cDNA was stored at −20°C until further analysis.

### Multiplexed RT-PCR

Previously amplified samples were loaded on a Fluidigm 96.96 standard chip following manufacturer’s instructions. All primers/probes used for the gene mix are TaqMan gene expression assays (Table S1 in Supplementary Material) and have been qualified on Human PBMCs and lymphocyte subsets following the method previously described (29). Gene selection was made according to previous analysis on Microarray of HIV-infected children (data not shown), literature, online gene banks, and biological queries. The sorting experiments and BioMark experiments were randomized to include a mix of HC, HIV, R, and NR patient samples so as not to bias the data toward one group by batch effects. Analysis was performed using Fluidigm Real-Time PCR Analysis software and “Multiple Chip Run” analysis mode. Cycle threshold values (Ct) were corrected according to the number of cells sorted if less than 500 according to the following calculation: *Y*/*X* = 67.5/500 (where *X* = actual number of cells sorted and *Y* = cDNA equivalent loaded onto BioMark chip). The dilution factor (*n*) was then calculated as *n* = 67.5/*Y* and log2(*n*) was subtracted from the Ct value to obtain Corrected Ct (cCt). Expression threshold (Et) values were calculated using the formula: Et = 40 − cCT, and Et was used for all downstream analysis. To verify consistency between individual BioMark runs, Et variance in B cells was calculated on the full set of genes. Housekeeping genes (*GAPDH, CD74*) included in our panel showed a low variation (<0.1 score) across all samples in both PBMCs and sorted B cell subsets (not shown).

### Enzyme-Linked Immunosorbent Assay (ELISA)

Plasma BAFF titers were measured as previously described (30). Briefly, plasma samples were diluted 1:1 and run in duplicate with 50 μL/well added to ELISA plates for human BAFF (R&D Systems).

### Bioinformatics and Statistical Analysis

Data were analyzed using Fluidigm SingulaR (SingulaR analysis toolset 3.0) package loaded on R (software R 3.0.2 GUI 1.62). We performed outlier identification analysis following manufacturer’s instructions (Singular Analysis Toolset User Guide) on the whole dataset by cell subset and removed outliers from subsequent analysis. ANOVA was used to identify differentially expressed genes (DEGs), and interplay between cell subsets or patient groups was assessed through fold increase of the averages. Inter-individual differences and outliers were analyzed by SingulaR. The “mixOmics” package (Omics data integration project) for R was used as previously described (31). Pearson or Spearman correlation plots were generated with Prism 6.0 (GraphPad) after performing kolmogorov-smirnov normality test to determine distribution of the data. Statistical differences between postvaccination (T1) and prevaccination (T0) gene expression were determined by Wilcoxon matched paired test, and volcano plot was generated in Prism 6.0.

## Results

### Perturbed Gene Expression in Memory B Cells Persists in HIV-Infected Children Despite Effective ART and Normal B Cell Frequency

In order to characterize the B cell compartment of vertically HIV-infected children under ART and stable viral control, we assessed frequencies of total B cells and B cell subsets by flow cytometry. No differences in frequencies were found between HIV-infected and HC groups (Figures 1B,C).

To evaluate the B cell compartment at the transcriptional level, we performed multiplexed RT-PCR of a panel of 96 genes (Table S1 in Supplementary Material) by Fluidigm Biomark™ in purified B cells from prevaccination samples. Principal component analysis (PCA) and hierarchical cluster analysis confirmed expected heterogeneity between memory subsets (AM and RM), and IgD^+^CD27^−^ (total naive) and DN subsets in HC and HIV (Figures S2A and S3 in Supplementary Material). The greatest transcriptional variation was found between RM and the other three subsets in both HIV and HC participants, especially between RM and AM (77 DEGs in HIV and 23 DEGs in HC) marked by overall lower gene expression in RM (Figure S2B in Supplementary Material).

As shown in Figure S2B in Supplementary Material, all 23 DEGs identified by the comparison of RM to AM transcripts in HC are also present in HIV-infected individuals. To better understand the biological context of genes identified by differential expression analysis between RM and AM, we performed gene set enrichment analysis (GSEA) of preranked gene lists using published blood transcription modules as gene sets (32). These genes are mainly involved in regulation of lymphocyte activation and leukocyte proliferation (*CD28, PILRB, FOXO3, CD38, STAT5A, ABCB1, CD40L*), suggesting common intrinsic gene expression patterns characterizing AM in both HIV and HC. However, 54 additional DEGs were identified in the HIV-infected group when comparing gene transcripts present in RM and AM. These genes include additional immune activation and lymphocyte proliferation genes (*CD86, CAV1, CAMK4, TNFSF13, BTLA, MTOR*) as well as genes involved in the inflammatory response (*CYBB, NOD2, MYD88, IL10, CCR2*), type I Interferon signaling (*IFIT2, MX1, STAT1*) and response to virus (*APOBEC3G, BST2, TRIM 5*), all with significantly higher expression in AM compared to RM. Overall, the lower gene expression found in RM compared to AM may suggest that they are in a quiescent phase.

**Figure 2:**
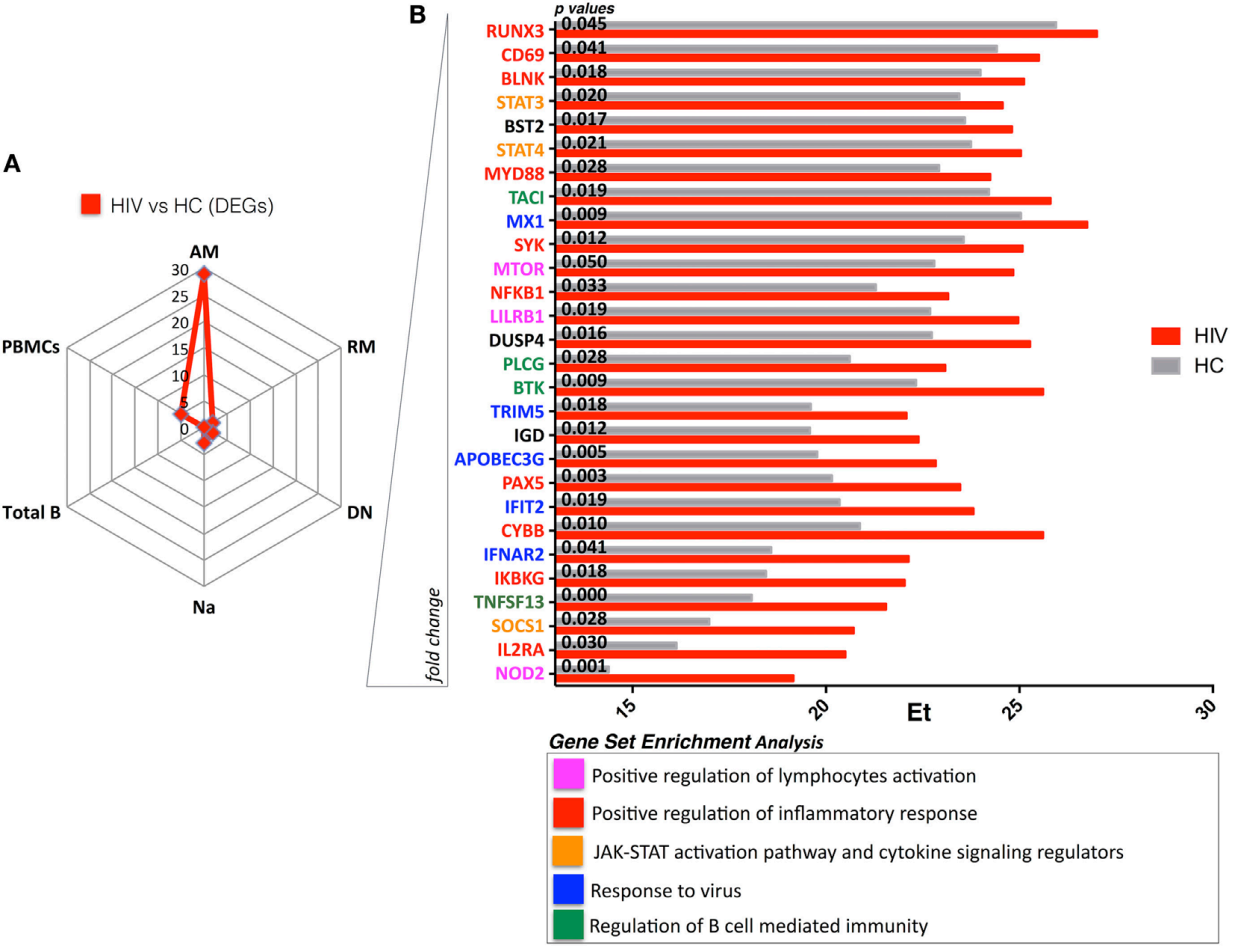
HIV present higher expression of genes involved in immuneactivation and inflammation in activated memory (AM) B cells despite effective antiretroviral therapy (ART) and long-term viral suppression. Graphs in panels **(A,B)** show comparisons in gene expression between healthy control (HC) and HIV. **(A)** Spider plot shows number of differentially expressed genes (DEGs) for all the subsets and total peripheral blood mononuclear cells (PBMCs). Box plots in panel **(B)** show gene expression averages from DEGS resulting in AM between HIV and HC (gene ranking defined by fold change). In this figure, *p*-values resulting from ANOVA analysis are shown. Color-labeled genes are defined according gene set enrichment analysis (performed by genemania.org) as described in the legend.

Next, we compared gene expression of each sorted B cell subset between HIV and HC to evaluate persistent defects in HIV infection despite viral control. Our results show that AM B cells clearly contrasted with 28 DEGs between HIV and HC (Figure 2A). Indeed, in this specific subset, already shown to dominate the HIV specific immune response in chronically infected adults (24), the DEGs showed higher expression in HIV compared to HC. Interestingly, this was not the case in PBMC and other sorted B cell subsets where few DEGs were identified in comparisons between HIV and HC: PBMC (5 DEGs), total B cells (0 DEGs), DN (2 DEGs), RM (2 DEGs), and total naive B cells (3 DEGs) (Figure 2A). GSEA analysis showed that genes expressed at higher levels in HIV compared to HC were mainly involved in inflammatory response and immune activation (*NOD2, IL2RA, SOCS1, IKBKG, CD69, CYBB, MYD88*) (Figure 2B; Figure S4 in Supplementary Material). Enrichment of *NOD2* (fivefold) and *IL2RA* (fourfold) was found in AM from HIV compared to HC. *NOD2* is mainly involved in signal transduction and activation of nuclear factor kappa-B during inflammatory responses, and the *IL2RA* is part of the IL-2 receptor complex and is involved in activation and proliferation of the cell after an external stimulus. Other genes involved in response to HIV entry (*APOBEC3G, TRIM5*) and positive regulation of B cell-mediated immunity (*BTK, TNSF13*) were also higher in AM of HIV compared to HC, suggesting that underlying activation in this cell subset persists despite effective ART and long-term viral control.

### B Cell Gene Expression Profiles in HIV-Infected Children with Differing Response to H1N1 Vaccine Antigen

**Figure 3:**
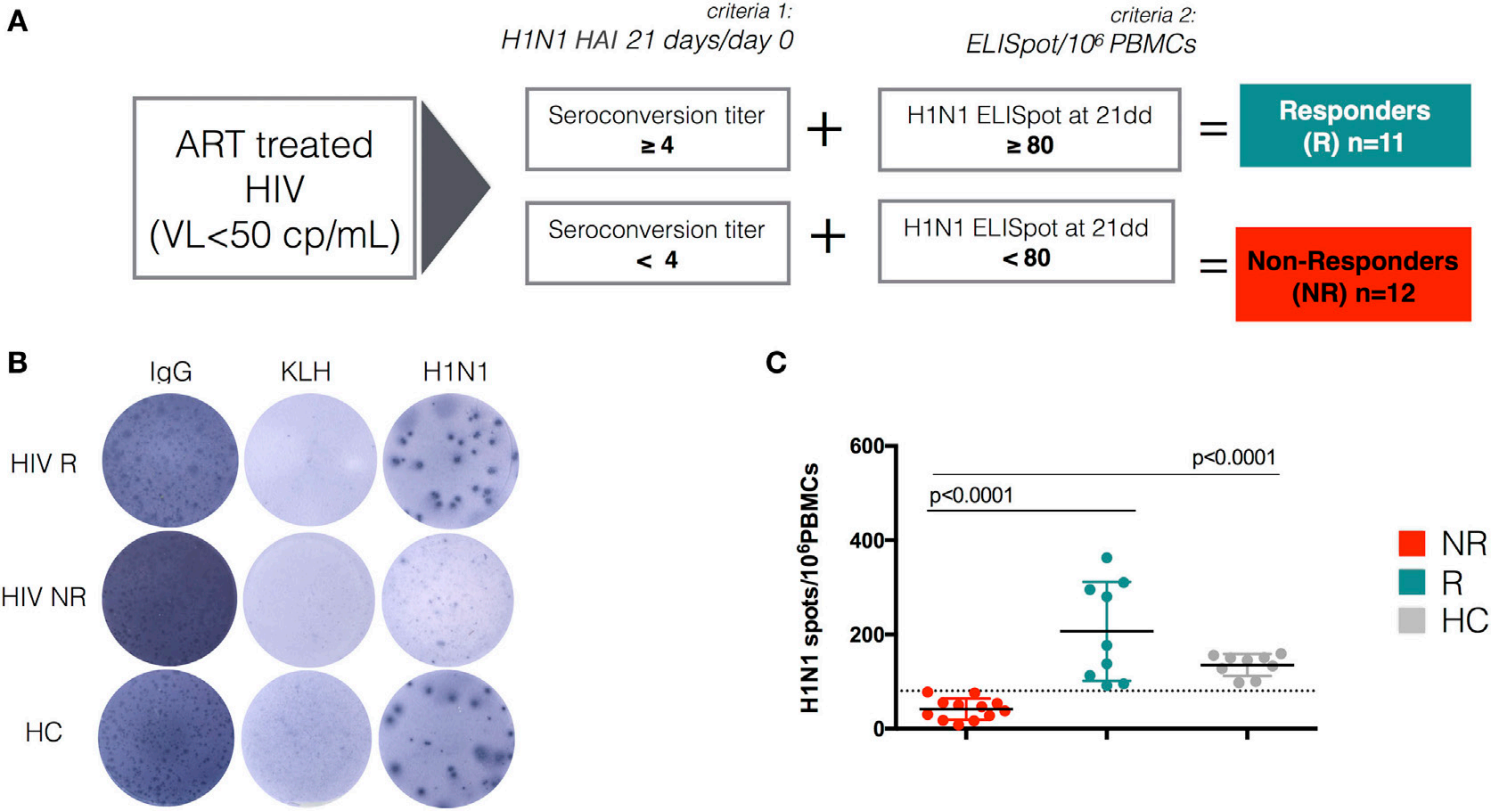
H1N1 response after trivalent inactivated influenza in HIV and perturbation of the memory compartment among non-responders. **(A)** The flow chart describes the criteria of selection used to define responders and non-responders to trivalent-inactivated influenza vaccination (TIV) among HIV-infected children. As a first criteria of selection, all patients were selected according to the fold increase of H1N1 hemagglutination inhibition (HAI) titer. Patients responding or not responding to the first selection criteria were further selected according to H1N1 ELISpot response. Selected healthy donors responded to the vaccination and met both criteria of selection. Representative ELISpot assay **(B)** and scatter dot plot **(C)** show ELISpot data for H1N1.

To determine how phenotype and transcriptional data associated with the ability of enrolled participants to respond to TIV, we applied two selection criteria (serology and Elispot) for separating study participants into responders (R) and NR (Figure 3A–C). The HIV-infected group contained approximately equal numbers of participants identified as R and NR, while all participants in the HC group were characterized as R. In agreement with our previous report (4), we found higher frequencies of IgD^−^CD27^−^ (DN) in NR compared to HC (Figure S5A in Supplementary Material). We also observed similar frequencies of class switched CD27^+^ memory B cells (CD19^+^CD27^+^IgD^−^) among the groups (HC, NR, and R) (Figure S5A in Supplementary Material); however, AM were significantly higher in NR compared to both R and HC (Figure S5B in Supplementary Material).

At a transcriptional level, intersubset analysis comparing AM and RM revealed fewer DEGs in R than NR due to overall higher gene expression in the RM subset from R (Figure S6A in Supplementary Material). We further noted that although most of the DEGs in the total HIV group were present in the comparison between NR and HC (47 DEGs) (Figure S6A and Table S2 in Supplementary Material), only 20 additional DEGs were identified between HIV R and HC (AM vs. RM).

Next, we performed GSEA on DEGs within AM from comparisons between HIV-infected participants (both R and NR separately) and HC. This analysis showed enriched pathways in positive regulation of apoptotic process (*FAS, BAX, PILRB*), B cell activation, and Fc receptor signaling (*BATF, FYN, PLCG1, CD27, CD28*) in HIV (Figures S6B,C and Table S3 in Supplementary Material). Collectively, gene expression data from AM B cells demonstrate that this subset, which has been shown to accumulate in individuals with HIV infection (14, 33), displays a distinct transcription profile compared to HC independent of TIV response.

### Distinct Prevaccination Gene Expression Patterns in RM from HIV-Infected Children Responding to H1N1

**Figure 4:**
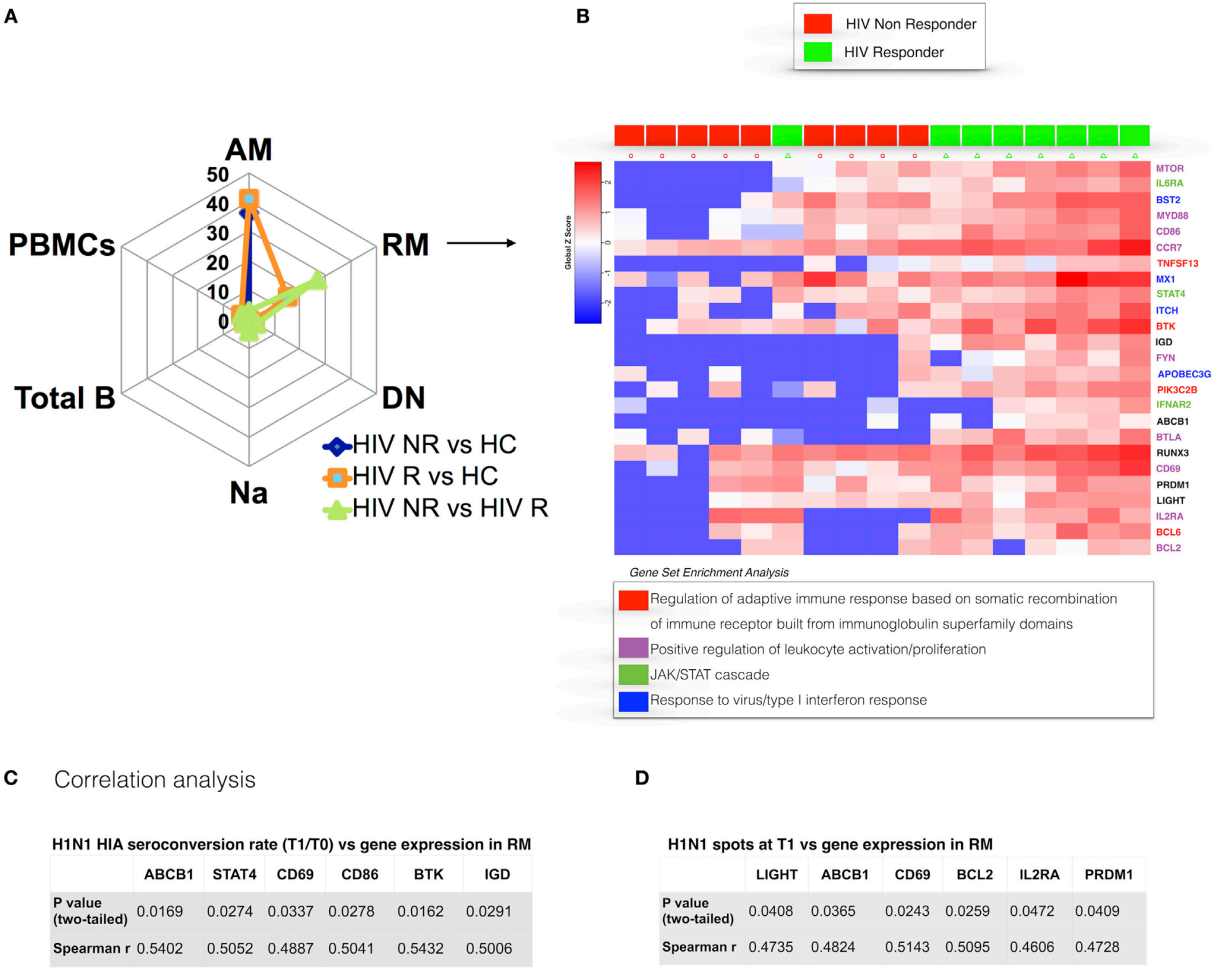
Prevaccination gene signatures in RM B cell subset discriminate HIV-infected R and non-responder (NR). **(A)** Spider plot shows number of differentially expressed genes (DEGs) for all the subsets and total peripheral blood mononuclear cells (PBMCs). **(B)** Heatmap shows gene expression in R and NR. Colored genes’ names refer to gene set enrichment analysis (GSEA) legend. In panels **(C,D)**, correlation between gene expression in resting memory and H1N1-seroconversion **(C)** and ELISpot at T1 **(D)** are shown. *p* and *r* values show results from correlation analyses (Pearson or Spearman tests for parametric and non-parametric data, respectively).

Our analysis of RM identified 25 genes that were differentially expressed between NR and R in HIV participants prior to vaccination with TIV (Figure 4A). RM from NR exhibited overall lower gene expression compared to HC and R. DEGs from this analysis, which were expressed higher in R, are directly involved in regulation of the adaptive immune response through somatic recombination from the immunoglobulin superfamily domain [*TNFSF13*(APRIL), *BTK*], leukocyte activation and BCR signaling pathways (*MTOR, FYN, CD86*). As shown in Figure 4B, genes involved in the *JAK/STAT* signaling cascade (*STAT4, IL6R, IFNAR*) and the closely related type I interferon response (*IFNAR2, MX1*) were higher in R. In addition, *PRDM1* (BLIMP1), able to induce B cell differentiation into plasma cells after encountering Ag (34), was higher in RM of R compared to NR. Collectively, these results show that the RM B cell subset, crucial for potent and specific adaptive immune responses, exhibits a distinct prevaccination transcriptional profile in HIV-infected participants who will mount an effective response to H1N1.

The gene set found to be different between NR and R was further analyzed for differences between pre vaccination (T0) and post vaccination (T1) gene expression in RM. Paired analysis revealed that *PRDM1* (BLIMP1) was significantly reduced at T1 when compared to T0 in HIV (*p* = 0.0039, median difference = −7.52) (Figure S7A in Supplementary Material). This longitudinal reduction was strongly confirmed in R with all R showing a reduction of *PRDM1* at T1 (*p* = 0.0001, median difference = −8.9), whereas significance was lost when only NR were taken into account (Figure S7B in Supplementary Material).

To further dissect the relationship of clinical (i.e., serological) markers of response to H1N1 and gene expression patterns, we performed pairwise correlation analysis using the two datasets. We confirmed the findings from differential gene expression analysis of prevaccination RM and found a positive correlation of *BTK* expression in RM at T0 and H1N1 seroconversion (HAI H1N1 Titer T1/T0) (Figure 4C) and H1N1 ELIspot at T1 (Figure 4D). Additional genes actively involved in proliferation and lymphocyte activation correlated with vaccine response in terms of H1N1 seroconversion (*CD69, CD86*) and H1N1 ELISpot at T1 (*CD69*). Interestingly, genes involved in inhibition of the apoptotic process (*LIGHT, BCL2*) showed positive correlations with H1N1 ELISpot at T1. Overall, these results demonstrate that the memory B cell compartment is highly impacted by HIV infection and suggest that an activated profile of specific genes in RM is required to maintain a normal adaptive response in HIV-infected patients.

We further asked whether gene expression analysis was correlated to measurements of plasma biomarkers or cell surface molecule expression encoded by the corresponding genes. To do so, we correlated gene expression data with plasma levels of BAFF (B cell activating factor) and with IL-21R^+^ B cell frequency, two molecules already shown to be involved in the immune response against H1N1 after vaccination (4, 30). Interestingly, *TNFSF13B* (BAFF) gene expression in RM was positively correlated with plasma BAFF levels at the time of vaccination (Figures S8A,C in Supplementary Material). Further, gene expression of *IL21R* in sorted RM was positively correlated with the expression of IL-21R as analyzed by flow cytometry (Figures S8B,D in Supplementary Material). These data show that transcriptional data may provide a functional correlate in specific molecules involved in the B cell memory response and maintenance over time.

## Discussion

This study represents the first evaluation of gene expression patterns in B cell subsets, total B cells and PBMCs in the field of pediatric HIV infection and in the context of immune responses to H1N1 antigen post-TIV. In the field of vaccinomics, systems biology tools have lately generated exciting data revealing molecular mechanisms of immunity induced by vaccination and correlates of protection in order to predict the vaccine efficacy in healthy adults (35, 36). However, as recently shown, the influence of age on gene expression patterns should be taken into account when interpreting systems biology data (37). Additionally, gene signatures identified in healthy adults and in the heterogeneous pool of PBMCs (38, 39) are not directly applicable to pediatric studies (40), and most likely not even in patients affected by chronic conditions such as HIV infection (41). Therefore, we believe that gene expression patterns identified in specific cell subsets may be crucial to investigate the dynamic of vaccine response in HIV-infected children.

In the present study, the analysis of gene expression from purified B cell subsets showed that perturbations in memory B cells persist in HIV-infected children despite stable and long-term virological control. Our results suggest that in these patients, the recovery achieved in overall B cell frequencies is not accompanied by recovery of gene expression and B cell function. We identified clear-cut differences in gene signatures between AM B cells of HIV-infected children and their healthy peers. B cell subsets between study groups were skewed in AM of HIV-infected children toward hyperexpression of genes involved in immune exhaustion/inflammation (*CYBB, MYD88, NOD2, IL2RA*) and apoptosis (*SOCS1, RUNX3*). The immune activation and exhaustion pattern, hereby confirmed at a transcriptional level in this particular subset of B cells, may play a key role in the “inflamm-aging” process which leaves ART-treated HIV-infected patients vulnerable to increased risk of non-AIDS defining comorbidities such as malignancies and cardiovascular diseases (42, 43). Indeed, despite the advent of ART which has dramatically increased life expectancy, non-AIDS defining malignancies are still increasing in ART-treated and virologically controlled HIV-infected children (44–46). The AM B cell subset was previously described to be enriched in HIV (47), to be prone to functional “exhaustion,” and to dominate HIV-specific responses (24). Furthermore, it has been recently reported in adults that signs of chronic inflammation persist over time even when treatment is started during acute infection (48). It is still unknown whether antiretroviral regimens may differentially impact B cell gene transcriptional patterns. Although in the present study, no differences in terms of ART regimen were found between the study groups (responders and not responders), these specific effects should be addressed in future investigations and in larger cohorts. Other differences between ART-treated HIV and uninfected children have emerged through comparison of B cell subsets within participant groups (see Figure S2B in Supplementary Material). However, we believe that transcriptional analysis of rare and still biologically ill-expanded cell subsets, such as DN and AM (47, 49), would benefit more from an unbiased whole transcriptome approach (e.g., RNA Seq) on sorted subsets and after *in vitro* or *in vivo* stimulation in order to provide more definitive results.

Despite this limitation, in line with our previous report (27) and together with findings reported herein, the perturbation of the AM subset may underlie mechanisms of premature aging of the immune system and impaired ability of HIV-infected patients to respond to vaccinations and to maintain a long-term immune response (50).

Although limited by the small sample size, gene expression data from RM B cells, revealed a 25 gene signature that distinguished responders and NR to H1N1. Interestingly, these data were derived from samples collected *before* vaccination. This observation may suggest that in the context of HIV infection, RM B cells, which provide secondary, potent and specific immune responses (51) need to present a specific gene expression pattern in order to provide an effective response to vaccination. Most of the genes involved in the signature are directly involved in the B cell receptor gene signaling cascade and in B cell development (*APRIL, BTK, PI3K, MTOR, BST2*), suggesting that a lower expression of these genes may contribute to a reduced Ab production upon Ag-recall responses. These results are in line with recent data suggesting that modules of genes related to B cell and plasmablasts may be crucial indicators and biomarkers of vaccine induced immunogenicity and protection (21). Although our study was mainly focused on prevaccination signature of response in HIV-infected patients, we performed longitudinal analysis to investigate differences in gene expression of RM from samples collected at 21 days after vaccination (T1) compared to baseline (T0). Expression of *PRDM1* (BLIMP1), a transcriptional repressor that drives terminal differentiation into plasma cells was found higher in RM of HIV responders at baseline and was significantly reduced at T1 when compared to T0 in HIV and particularly in R (Figure S7 in Supplementary Material). Reduction of *PRDM1* may represent the resting phase of Ag specific B cells after migration to the germinal centers, class switch recombination, and somatic hypermutation (34, 52). Another consideration is that earlier timepoints (24 h to 1 week) after immunization or after re-exposure to the Ag will need to be tested in order to confirm this hypothesis and define the genes’ activation programs which orchestrate memory B cell responses in HIV-infected children. Indeed in recent studies early changes of genes enriched in B cells, plasmablasts and immunoglobulins after administration of the RTS,S/AS01 malaria vaccine in healthy malaria-naive adults, were found to be related to vaccine Ab production and cell-related immunogenicity (19).

Our data on RM transcriptional signatures revealed that H1N1 responders expressed higher JAK-STAT cascade genes (*MX1, IFNAR, STAT4*). These findings are consistent with previous reports showing that STAT genes are crucial in the differentiation of RM B cells induced by IL-21 (53). In this context and following a similar experimental approach as this, we recently reported that *IL21* gene expression from prevaccination purified peripheral T follicular helper cells (pTfh) after *in vitro* stimulation, is an indicator of vaccine response (54).

The present study identified predictive correlates of seroconversion following immunization using pair-wise correlation analysis between individual gene expression data and serological data. In RM, *BTK*, involved in B cell development, and *CD86*, a lymphocyte activation gene, showed significant positive correlations with H1N1 seroconversion after vaccination in HIV supporting the hypothesis that gene signatures in purified RM B cells at the time of immunization may predict the ability of HIV-infected children to respond to vaccinations. Taken together these findings suggest that specific gene signatures in cell subsets directly involved in Ab production and response to Ag (pTfh and RM B cells) are needed to provide an efficient immune response and are altered in HIV infection.

This experimental approach, based on a targeted gene selection (*n* = 96) rather than unbiased whole transcriptome sequencing, illustrates the benefits of analysis of purified cell subsets. The increased specificity resulting from this approach is important, considering the observed phenotypic alterations in immune cells from HIV-infected patients. We believe that these data provide a strong rationale to warrant future larger studies that can expand and validate these findings.

## Ethics Statement

Written informed consent was obtained from all subjects or parents/guardians and the local Institutional review board approved the study.

## Author Contributions

NC, LA, SuP, PP, and SP conceived the study and designed the experiments. NC and LA performed the experimental procedures. NC drafted the first version of the article. All authors participated in writing, review and editing of the article. NC, LA, BI, and SR performed statistical analysis and bioinformatics. Supervision and resources were provided by PR, PP, and SP.

## Conflict of Interest Statement

The authors declare that the research was conducted in the absence of any commercial or financial relationships that could be construed as a potential conflict of interest.

## Supplementary Material

Figure S1 Experimental design. Cartoon in panel **(A)** depicts design of the study. Peripheral blood mononuclear cells (PBMCs) were collected at the time of vaccination (T0) and 21 days after vaccination (T1). At both timepoints, hemagglutination inhibition assay (HIA), and H1N1 ELISpot were performed. Briefly, in panel **(B)** PBMCs stored in liquid nitrogen were thawed and stained for surface molecules and analyzed by flow cytometry (ARIA II cs). Equal number (500 cells) of cells from 5 subsets and total PBMCs from unstimulated samples were sorted as depicted in the sorting strategy into tubes previously coated with specific polymerase chain reaction (PCR) buffer. Data, collected through Fluidigm Real Time PCR analysis software was then analyzed through Fluidigm SingulaR (SingulaR analysis toolset 3.0) package, loaded on R (software R 3.0.2 GUI 1.62). Data were later used for mixOmics on R (mixOmics package) and for gene set enrichment analysis (GSEA).

Figure S2 Gene expression patterns of memory B cell subsets rather than B cell frequencies differentiates virally controlled HIV-infected children from their healthy peers. **(A)** Principal component analysis (PCA) shows segregation among the four different B cell subsets in healthy controls (HC) and HIV. JMP©, SAS^®^ has been used to produce the PCA. **(B)** Venn diagram shows differentially expressed genes (DEGs) found when gene expression of activated memory (AM) and resting memory (RM) are compared between HIV and HC. Only ANOVA analyses with a *p* value < 0.05 are shown. Genes marked with the asterisk show *p* values ≤ 0.01. All 23 DEGs found in HC were overlapping in HIV (light gray box). 54 additional DEGs were found in HIV only (dark gray box).

Figure S3 Differential inter subset analysis. Heatmap of intersubset analysis in healthy controls (HC) **(A)** HIV **(B)**, R **(C)**, and NR **(D)**. Heatmap analyses were generated by singular analysis toolset after identification and removal of outliers.

Figure S4 Gene set enrichment analysis from differentially expressed genes resulting from activated memory in HIV vs. healthy controls (HC). Gene Set Enrichment Analysis in the graph was generated by genemania cytoscape app (genemania.org).

Figure S5 Scatter dot plot in panel (A) shows frequencies of total B cells (live, CD10^−^, CD20^+^); and among B cells: double negative (CD27^−^, IgD^−^), total naive (CD27^−^, IgD^+^), class switched CD27^+^ memory B cells (CD27^+^, IgD^−^), tissue like (CD27^−^, IgD^−^, CD21^−^). In panel **(B)** activated memory (CD27^+^, IgD^−^, CD21^−^) and resting memory (CD27^+^, IgD^−^, CD21^+^) (two tailed Mann–Whitney test for comparisons) are shown.

Figure S6 Activated memory (AM) vs. resting memory (RM) differentially expressed genes (DEGs) in healthy controls (HC), R and NR. Venn Diagram in panel (A) shows DEGs found when gene expression of AM and RM are compared between HIV R, HIV NR, and HC. Only ANOVA analysis with a *p* value < 0.05 are shown. **(B,C)** gene set enrichment analysis of DEGs derived from activated memory vs. resting memory of HIV NR vs. HC **(B)** and HIV R vs. HC **(C)** are shown.

Figure S7 Post vaccination (T1) *PRDM1* (*BLIMP1*) gene expression is reduced in resting memory (RM) of HIV-infected children compared to baseline (T0). In panel **(A)**, volcano plot shows differences in gene expression between T1 and T0 in selected genes from sorted RM. In panel **(B)** paired analysis in R (green) and NR (red) of PRDM1 are shown. *P*-Values and median differences derive from Wilcoxon paired t test performed by graphpad (prism 6.0).

Figure S8 IL21R and BAFF (*TNFSF13B*) gene expression in resting memory (RM) correlates with surface molecules and plasma levels. In panel **(A)** heatmap analysis shows *r* values resulting from pairwise Spearman correlations between the aforementioned observations. In **panels (B–D)**, correlation dot plots of the afore mentioned analysis are shown. In the graph gene expression resulting from RM is shown.

Table S1 The full list of genes, alias names and assay codes used for multiplexed principal component analysis are shown in the table.

Table S2 Differentially expressed genes (DEGs) between cell subsets in HIV, healthy controls (HC), R and NR. Table shows DEGs in all groups (HIV, HC, HIV NR, HIV R) when subsets were compared. In the table, cells are highlighted in orange when *p* values were <0.01 and not highlighted when *p* value <0.05. Outlined cells define an opposite relation compared to the one in the headline.

Table S3 Differentially expressed genes between study groups. Note: *genemania.org gene set enrichment analysis (GSEA)* In every graph presented throughout the paper, circles with diagonal lines define genes included in the GSEA. Its function is described legend below the graph. The solid circles represent genes resulted to be involved in the pathways. Networks among the genes are defined by colored lines, further explained by legends.

## Abbreviations

ART, antiretroviral therapy; BCR, B cell receptor; TIV, trivalent-inactivated influenza vaccination; LAIV, live attenuated influenza vaccination; PBMCs, peripheral blood mononuclear cells; RM, resting memory; AM, activated memory; DN, double negative; DEGs, differentially expressed genes; ANOVA, analysis of variance; HCs, healthy controls; HAI, hemagglutination inhibition; Ct, cycle threshold; Et, expression threshold; PCR, polymerase chain reaction; SPBS, sort-purified B cell subsets.

## References

[1] Amu S Ruffin N Rethi B Chiodi F. Impairment of B-cell functions during HIV-1 infection. AIDS (2013) 27(15):2323–34. 10.1097/QAD.0b013e328361a427 23595152

[2] Titanji K De Milito A Cagigi A Thorstensson R Grutzmeier S Atlas A Loss of memory B cells impairs maintenance of long-term serologic memory during HIV-1 infection. Blood (2006) 108(5):1580–7. 10.1182/blood-2005-11-013383 16645169

[3] Bamford A Hart M Lyall H Goldblatt D Kelleher P Kampmann B. The influence of paediatric HIV infection on circulating B cell subsets and CXCR5(+) T helper cells. Clin Exp Immunol (2015) 181(1):110–7. 10.1111/cei.12618 25737039 PMC4469160

[4] Cagigi A Rinaldi S Di Martino A Manno EC Zangari P Aquilani A Premature immune senescence during HIV-1 vertical infection relates with response to influenza vaccination. J Allergy Clin Immunol (2014) 133(2):592–4. 10.1016/j.jaci.2013.10.003 24290278

[5] Sallusto F Lanzavecchia A Araki K Ahmed R. From vaccines to memory and back. Immunity (2010) 33(4):451–63. 10.1016/j.immuni.2010.10.008 21029957 PMC3760154

[6] Rainwater-Lovett K Nkamba HC Mubiana-Mbewe M Moore CB Margolick JB Moss WJ. Antiretroviral therapy restores age-dependent loss of resting memory B cells in young HIV-infected Zambian children. J Acquir Immune Defic Syndr (2014) 65(5):505–9. 10.1097/QAI.0000000000000074 24326598 PMC3999252

[7] Sutcliffe CG Moss WJ. Do children infected with HIV receiving HAART need to be revaccinated? Lancet Infect Dis (2010) 10(9):630–42. 10.1016/S1473-3099(10)70116-X 20797645

[8] Cagigi A Cotugno N Giaquinto C Nicolosi L Bernardi S Rossi P Immune reconstitution and vaccination outcome in HIV-1 infected children: present knowledge and future directions. Hum Vaccin Immunother (2012) 8(12):1784–94. 10.4161/hv.21827 22906931 PMC3656066

[9] Rainwater-Lovett K Moss WJ The urgent need for recommendations on revaccination of HIV-infected children after successful antiretroviral therapy. Clin Infect Dis (2010) 51(5):634–5. 10.1086/655769 20684683

[10] Pallikkuth S Parmigiani A Silva SY George VK Fischl M Pahwa R Impaired peripheral blood T-follicular helper cell function in HIV-infected nonresponders to the 2009 H1N1/09 vaccine. Blood (2012) 120(5):985–93. 10.1182/blood-2011-12-396648 22692510 PMC3412336

[11] Pallikkuth S Pilakka Kanthikeel S Silva SY Fischl M Pahwa R Pahwa S. Upregulation of IL-21 receptor on B cells and IL-21 secretion distinguishes novel 2009 H1N1 vaccine responders from nonresponders among HIV-infected persons on combination antiretroviral therapy. J Immunol (2011) 186(11):6173–81. 10.4049/jimmunol.1100264 21531891 PMC3170914

[12] Boyd SD Jackson KJ. Predicting vaccine responsiveness. Cell Host Microbe (2015) 17(3):301–7. 10.1016/j.chom.2015.02.015 25766292

[13] Curtis DJ Muresan P Nachman S Fenton T Richardson KM Dominguez T Characterization of functional antibody and memory B-cell responses to pH1N1 monovalent vaccine in HIV-infected children and youth. PLoS One (2015) 10(3):e0118567. 10.1371/journal.pone.0118567 25785995 PMC4364897

[14] Cotugno N Douagi I Rossi P Palma P. Suboptimal immune reconstitution in vertically HIV infected children: a view on how HIV replication and timing of HAART initiation can impact on T and B-cell compartment. Clin Dev Immunol (2012) 2012:805151. 10.1155/2012/805151 22550537 PMC3328919

[15] Querec TD Akondy RS Lee EK Cao W Nakaya HI Teuwen D Systems biology approach predicts immunogenicity of the yellow fever vaccine in humans. Nat Immunol (2009) 10(1):116–25. 10.1038/ni.1688 19029902 PMC4049462

[16] Pulendran B Li S Nakaya HI. Systems vaccinology. Immunity (2010) 33(4):516–29. 10.1016/j.immuni.2010.10.006 21029962 PMC3001343

[17] Poland GA Kennedy RB McKinney BA Ovsyannikova IG Lambert ND Jacobson RM Vaccinomics, adversomics, and the immune response network theory: individualized vaccinology in the 21st century. Semin Immunol (2013) 25(2):89–103. 10.1016/j.smim.2013.04.007 23755893 PMC3752773

[18] Tan Y Tamayo P Nakaya H Pulendran B Mesirov JP Haining WN. Gene signatures related to B-cell proliferation predict influenza vaccine-induced antibody response. Eur J Immunol (2014) 44(1):285–95. 10.1002/eji.201343657 24136404 PMC3973429

[19] Kazmin D Nakaya HI Lee EK Johnson MJ van der Most R van den Berg RA Systems analysis of protective immune responses to RTS,S malaria vaccination in humans. Proc Natl Acad Sci U S A (2017) 114(9):2425–30. 10.1073/pnas.1621489114 28193898 PMC5338562

[20] Nakaya HI Wrammert J Lee EK Racioppi L Marie-Kunze S Haining WN Systems biology of vaccination for seasonal influenza in humans. Nat Immunol (2011) 12(8):786–95. 10.1038/ni.2067 21743478 PMC3140559

[21] Li S Rouphael N Duraisingham S Romero-Steiner S Presnell S Davis C Molecular signatures of antibody responses derived from a systems biology study of five human vaccines. Nat Immunol (2014) 15(2):195–204. 10.1038/ni.2789 24336226 PMC3946932

[22] Oberg AL McKinney BA Schaid DJ Pankratz VS Kennedy RB Poland GA. Lessons learned in the analysis of high-dimensional data in vaccinomics. Vaccine (2015) 33(40):5262–70. 10.1016/j.vaccine.2015.04.088 25957070 PMC4581898

[23] Tsang JS Schwartzberg PL Kotliarov Y Biancotto A Xie Z Germain RN Global analyses of human immune variation reveal baseline predictors of postvaccination responses. Cell (2014) 157(2):499–513. 10.1016/j.cell.2014.03.031 24725414 PMC4139290

[24] Kardava L Moir S Shah N Wang W Wilson R Buckner CM Abnormal B cell memory subsets dominate HIV-specific responses in infected individuals. J Clin Invest (2014) 124(7):3252–62. 10.1172/JCI74351 24892810 PMC4071400

[25] Cotugno N De Armas L Pallikkuth S Rossi P Palma P Pahwa S. Paediatric HIV infection in the ’omics era: defining transcriptional signatures of viral control and vaccine responses. J Virus Erad (2015) 1:153–8. 26807446 10.1016/S2055-6640(20)30507-0 PMC4721557

[26] Boyum A Isolation of mononuclear cells and granulocytes from human blood. Isolation of monuclear cells by one centrifugation, and of granulocytes by combining centrifugation and sedimentation at 1 g. Scand J Clin Lab Invest Suppl (1968) 97:77–89. 4179068

[27] Cagigi A Rinaldi S Santilli V Mora N C Manno E Cotugno N Premature ageing of the immune system relates to increased anti-lymphocyte antibodies (ALA) after an immunization in HIV-1-infected and kidney-transplanted patients. Clin Exp Immunol (2013) 174(2):274–80. 10.1111/cei.12173 23841754 PMC3828831

[28] Cagigi A Pensieroso S Ruffin N Sammicheli S Thorstensson R Pan-Hammarstrom Q Relation of activation-induced deaminase (AID) expression with antibody response to A(H1N1)pdm09 vaccination in HIV-1 infected patients. Vaccine (2013) 31(18):2231–7. 10.1016/j.vaccine.2013.03.002 23499520

[29] Dominguez MH Chattopadhyay PK Ma S Lamoreaux L McDavid A Finak G Highly multiplexed quantitation of gene expression on single cells. J Immunol Methods (2013) 391(1–2):133–45. 10.1016/j.jim.2013.03.002 23500781 PMC3814038

[30] Pallikkuth S Kanthikeel SP Silva SY Fischl M Pahwa R Pahwa S. Innate immune defects correlate with failure of antibody responses to H1N1/09 vaccine in HIV-infected patients. J Allergy Clin Immunol (2011) 128(6):1279–85. 10.1016/j.jaci.2011.05.033 21752440 PMC3229646

[31] Liquet B Le Cao KA Hocini H Thiebaut R A novel approach for biomarker selection and the integration of repeated measures experiments from two assays. BMC Bioinformatics (2012) 13:325 10.1186/1471-2105-13-325 23216942 PMC3627901

[32] Warde-Farley D Donaldson SL Comes O Zuberi K Badrawi R Chao P The GeneMANIA prediction server: biological network integration for gene prioritization and predicting gene function. Nucleic Acids Res (2010) 38:W214–20. 10.1093/nar/gkq537 20576703 PMC2896186

[33] Hu Z Luo Z Wan Z Wu H Li W Zhang T HIV-associated memory B cell perturbations. Vaccine (2015) 33(22):2524–9. 10.1016/j.vaccine.2015.04.008 25887082 PMC4420662

[34] Shapiro-Shelef M Lin KI McHeyzer-Williams LJ Liao J McHeyzer-Williams MG Calame K. Blimp-1 is required for the formation of immunoglobulin secreting plasma cells and pre-plasma memory B cells. Immunity (2003) 19(4):607–20. 10.1016/S1074-7613(03)00267-X 14563324

[35] Nakaya HI Pulendran B Vaccinology in the era of high-throughput biology. Philos Trans R Soc Lond B Biol Sci (1671) 2015:370. 10.1098/rstb.2014.0146 PMC4527391 25964458

[36] Ovsyannikova IG Salk HM Kennedy RB Haralambieva IH Zimmermann MT Grill DE Gene signatures associated with adaptive humoral immunity following seasonal influenza A/H1N1 vaccination. Genes Immun (2016) 17(7):371–9. 10.1038/gene.2016.34 27534615 PMC5133148

[37] Nakaya HI Hagan T Duraisingham SS Lee EK Kwissa M Rouphael N Systems analysis of immunity to influenza vaccination across multiple years and in diverse populations reveals shared molecular signatures. Immunity (2015) 43(6):1186–98. 10.1016/j.immuni.2015.11.012 26682988 PMC4859820

[38] Haining WN Wherry EJ. Integrating genomic signatures for immunologic discovery. Immunity (2010) 32(2):152–61. 10.1016/j.immuni.2010.02.001 20189480

[39] Gaucher D Therrien R Kettaf N Angermann BR Boucher G Filali-Mouhim A Yellow fever vaccine induces integrated multilineage and polyfunctional immune responses. J Exp Med (2008) 205(13):3119–31. 10.1084/jem.20082292 19047440 PMC2605227

[40] Nakaya HI Clutterbuck E Kazmin D Wang L Cortese M Bosinger SE Systems biology of immunity to MF59-adjuvanted versus nonadjuvanted trivalent seasonal influenza vaccines in early childhood. Proc Natl Acad Sci U S A (2016) 113(7):1853–8. 10.1073/pnas.1519690113 26755593 PMC4763735

[41] Virgin HW Wherry EJ Ahmed R. Redefining chronic viral infection. Cell (2009) 138(1):30–50. 10.1016/j.cell.2009.06.036 19596234

[42] Alcaide ML Parmigiani A Pallikkuth S Roach M Freguja R Della Negra M Immune activation in HIV-infected aging women on antiretrovirals – implications for age-associated comorbidities: a cross-sectional pilot study. PLoS One (2013) 8(5):e63804 10.1371/journal.pone.0063804 23724003 PMC3665816

[43] Volberding PA Deeks SG. Antiretroviral therapy and management of HIV infection. Lancet (2010) 376(9734):49–62. 10.1016/S0140-6736(10)60676-9 20609987

[44] Chiappini E Berti E Gianesin K Petrara MR Galli L Giaquinto C Pediatric human immunodeficiency virus infection and cancer in the highly active antiretroviral treatment (HAART) era. Cancer Lett (2014) 347(1):38–45. 10.1016/j.canlet.2014.02.002 24513180

[45] Alvaro-Meca A Micheloud D Jensen J Diaz A Garcia-Alvarez M Resino S. Epidemiologic trends of cancer diagnoses among HIV-infected children in Spain from 1997 to 2008. Pediatr Infect Dis J (2011) 30(9):764–8. 10.1097/INF.0b013e31821ba148 21494172

[46] Zangari P Santilli V Cotugno N Manno E Palumbo G Lombardi A Raising awareness of non-Hodgkin lymphoma in HIV-infected adolescents: report of 2 cases in the HAART era. J Pediatr Hematol Oncol (2013) 35(3):e134–7. 10.1097/MPH.0b013e318282cef5 23426000

[47] Moir S Fauci AS B-cell exhaustion in HIV infection: the role of immune activation. Curr Opin HIV AIDS (2014) 9(5):472–7. 10.1097/COH.0000000000000092 25023621

[48] Sereti I Krebs SJ Phanuphak N Fletcher JL Slike B Pinyakorn S Persistent, Albeit reduced, chronic inflammation in persons starting antiretroviral therapy in acute HIV infection. Clin Infect Dis (2017) 64(2):124–31. 10.1093/cid/ciw683 27737952 PMC5215214

[49] Moir S Fauci AS. B-cell responses to HIV infection. Immunol Rev (2017) 275(1):33–48. 10.1111/imr.12502 28133792 PMC5300048

[50] Palma P Rinaldi S Cotugno N Santilli V Pahwa S Rossi P Premature B-cell senescence as a consequence of chronic immune activation. Hum Vaccin Immunother (2014) 10(7):2083–8. 10.4161/hv.28698 25424820 PMC4186020

[51] Good KL Avery DT Tangye SG Resting human memory B cells are intrinsically programmed for enhanced survival and responsiveness to diverse stimuli compared to naive B cells. J Immunol (2009) 182(2):890–901. 10.4049/jimmunol.182.2.890 19124732

[52] Shaffer AL Lin KI Kuo TC Yu X Hurt EM Rosenwald A Blimp-1 orchestrates plasma cell differentiation by extinguishing the mature B cell gene expression program. Immunity (2002) 17(1):51–62. 10.1016/S1074-7613(02)00335-7 12150891

[53] Deenick EK Avery DT Chan A Berglund LJ Ives ML Moens L Naive and memory human B cells have distinct requirements for STAT3 activation to differentiate into antibody-secreting plasma cells. J Exp Med (2013) 210(12):2739–53. 10.1084/jem.20130323 24218138 PMC3832925

[54] de Armas LR Cotugno N Pallikkuth S Pan L Rinaldi S Sanchez MC Induction of IL21 in peripheral T follicular helper cells is an indicator of influenza vaccine response in a previously vaccinated HIV-infected Pediatric Cohort. J Immunol (2017) 198(5):1995–2005. 10.4049/jimmunol.1601425 28130496 PMC5322168

