# Supplementary figures and images for: Perturbation of B Cell Gene Expression Persists in HIV-Infected Children Despite Effective Antiretroviral Therapy and Predicts H1N1 Response
Source: Front Immunol. 2017 Sep 11;8:1083. doi: 10.3389/fimmu.2017.01083 (PMC5600985; doi:10.3389/fimmu.2017.01083)

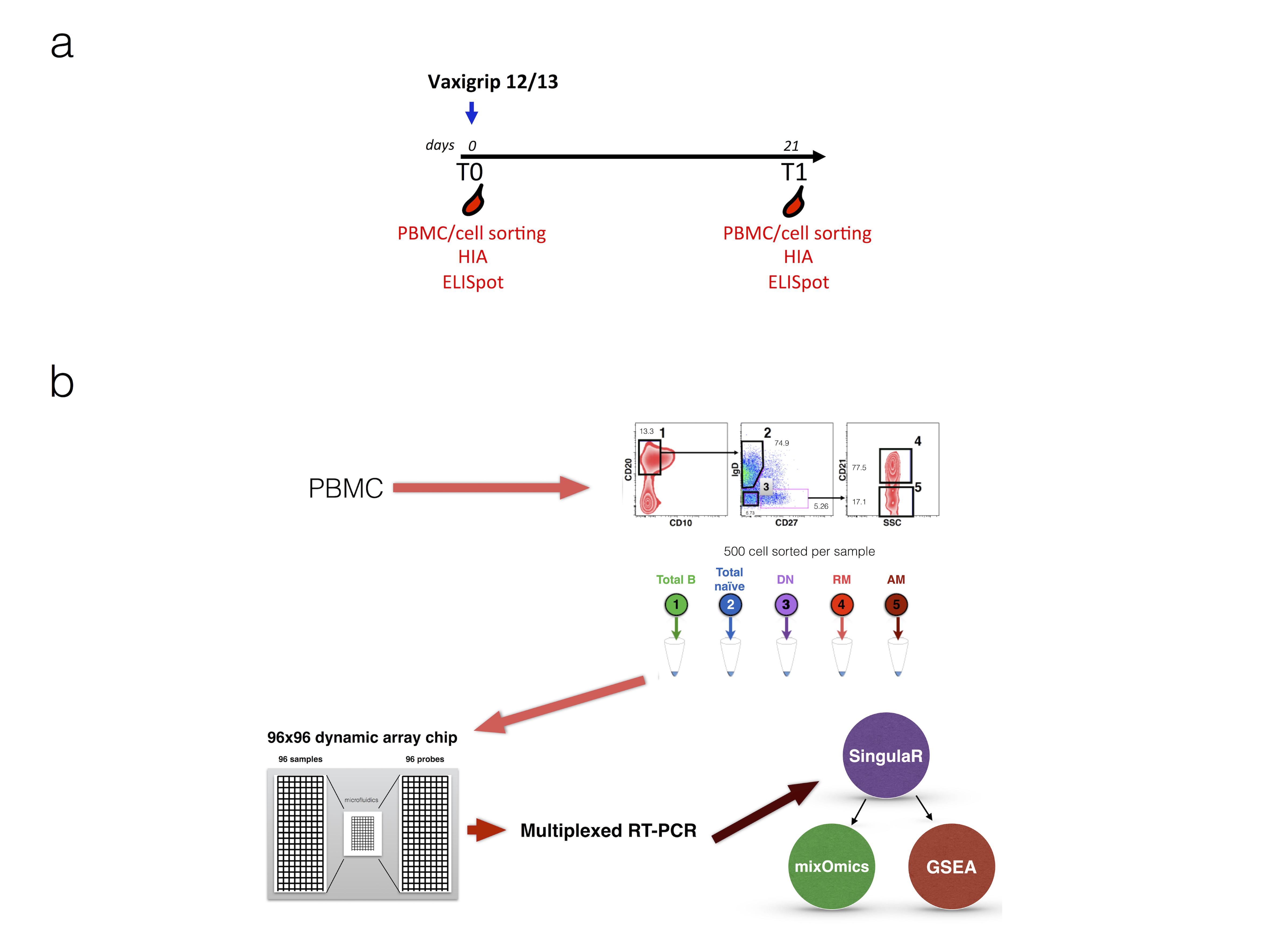

Supplement: Figure S1 — Experimental design. Cartoon in panel (A) depicts design of the study. Peripheral blood mononuclear cells (PBMCs) were collected at the time of vaccination (T0) and 21 days after vaccination (T1). At both timepoints, hemagglutination inhibition assay (HIA), and H1N1 ELISpot were performed. Briefly, in panel (B) PBMCs stored in liquid nitrogen were thawed and stained for surface molecules and analyzed by flow cytometry (ARIA II cs). Equal number (500 cells) of cells from 5 subsets and total PBMCs from unstimulated samples were sorted as depicted in the sorting strategy into tubes previously coated with specific polymerase chain reaction (PCR) buffer. Data, collected through Fluidigm Real Time PCR analysis software was then analyzed through Fluidigm SingulaR (SingulaR analysis toolset 3.0) package, loaded on R (software R 3.0.2 GUI 1.62). Data were later used for mixOmics on R (mixOmics package) and for gene set enrichment analysis (GSEA). [file image_1.tiff]

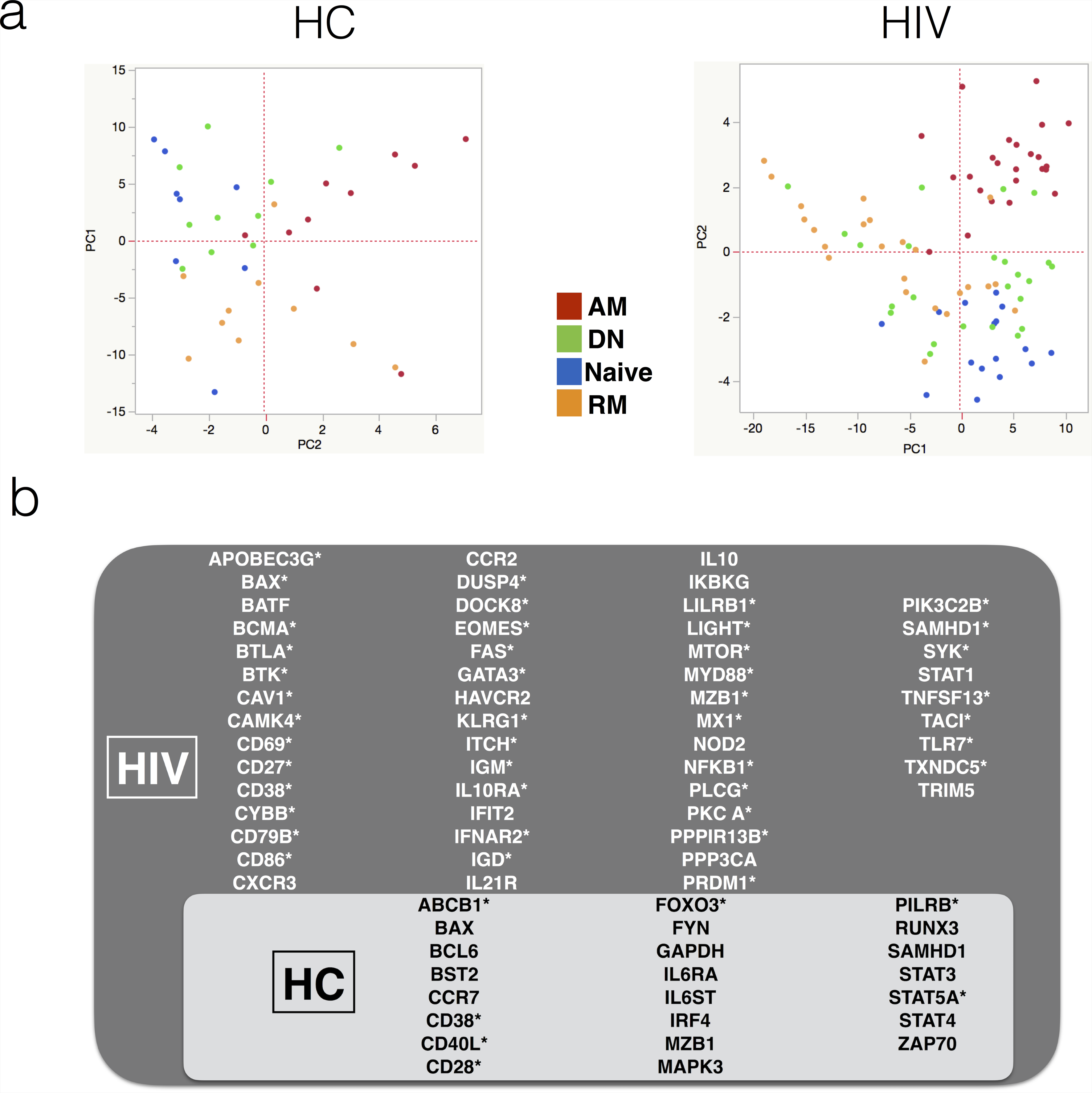

Supplement: Figure S2 — Gene expression patterns of memory B cell subsets rather than B cell frequencies differentiates virally controlled HIV-infected children from their healthy peers. (A) Principal component analysis (PCA) shows segregation among the four different B cell subsets in healthy controls (HC) and HIV. JMP©, SAS® has been used to produce the PCA. (B) Venn diagram shows differentially expressed genes (DEGs) found when gene expression of activated memory (AM) and resting memory (RM) are compared between HIV and HC. Only ANOVA analyses with a p value < 0.05 are shown. Genes marked with the asterisk show p values ≤ 0.01. All 23 DEGs found in HC were overlapping in HIV (light gray box). 54 additional DEGs were found in HIV only (dark gray box). [file image_2.tiff]

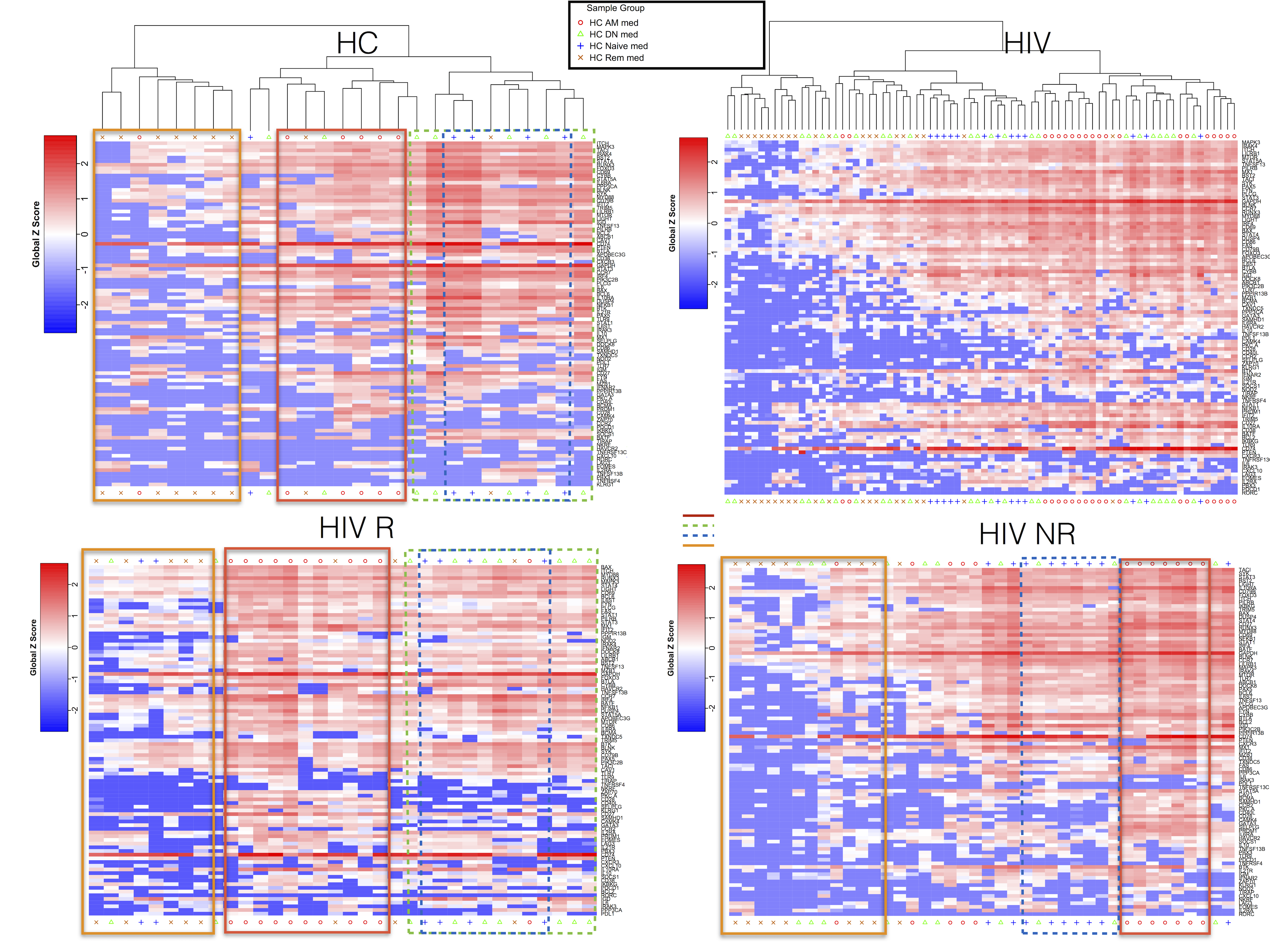

Supplement: Figure S3 — Differential inter subset analysis. Heatmap of intersubset analysis in healthy controls (HC) (A) HIV (B), R (C), and NR (D). Heatmap analyses were generated by singular analysis toolset after identification and removal of outliers. [file image_3.tiff]

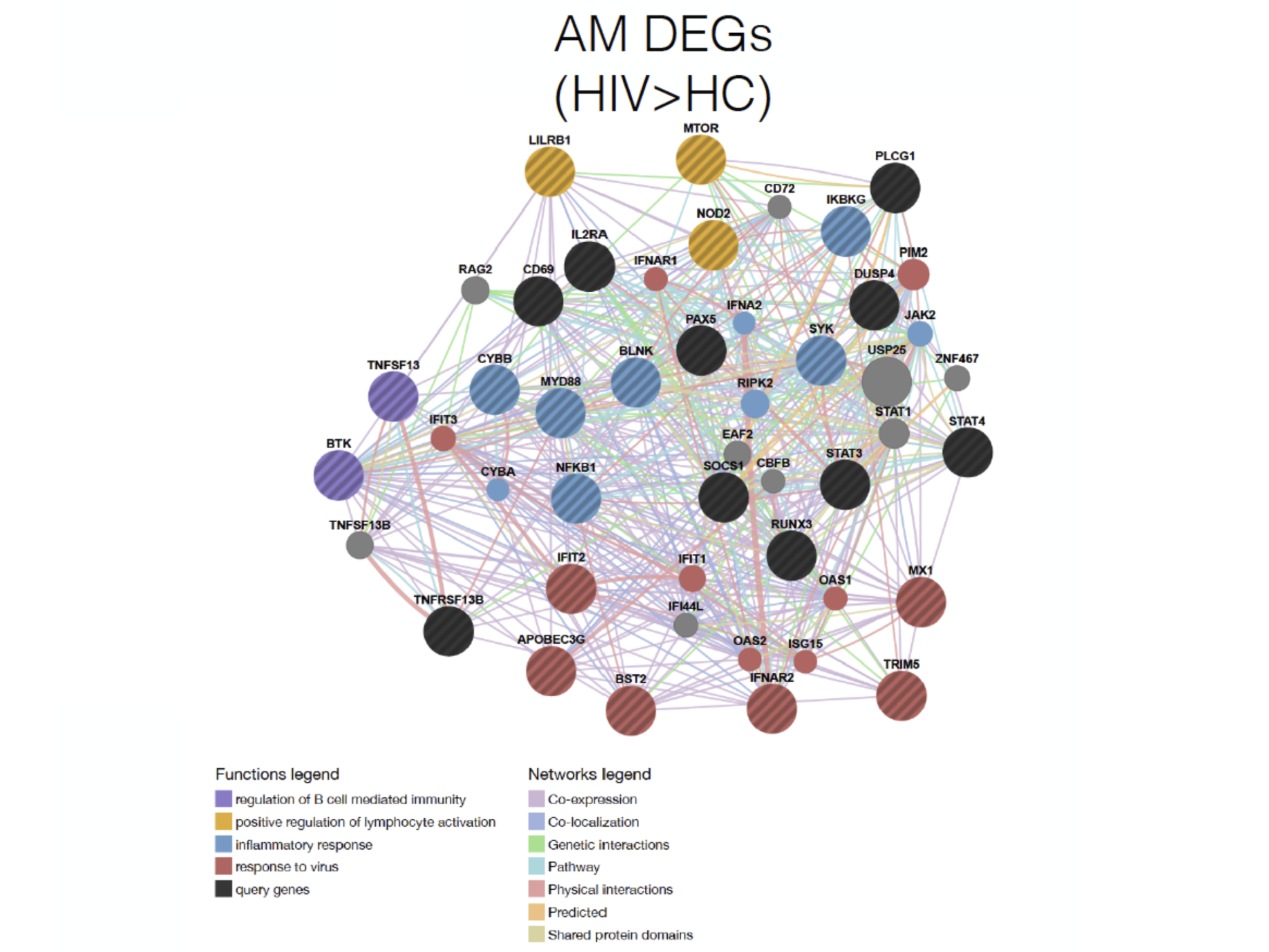

Supplement: Figure S4 — Gene set enrichment analysis from differentially expressed genes resulting from activated memory in HIV vs. healthy controls (HC). Gene Set Enrichment Analysis in the graph was generated by genemania cytoscape app (genemania.org). [file image_4.tiff]

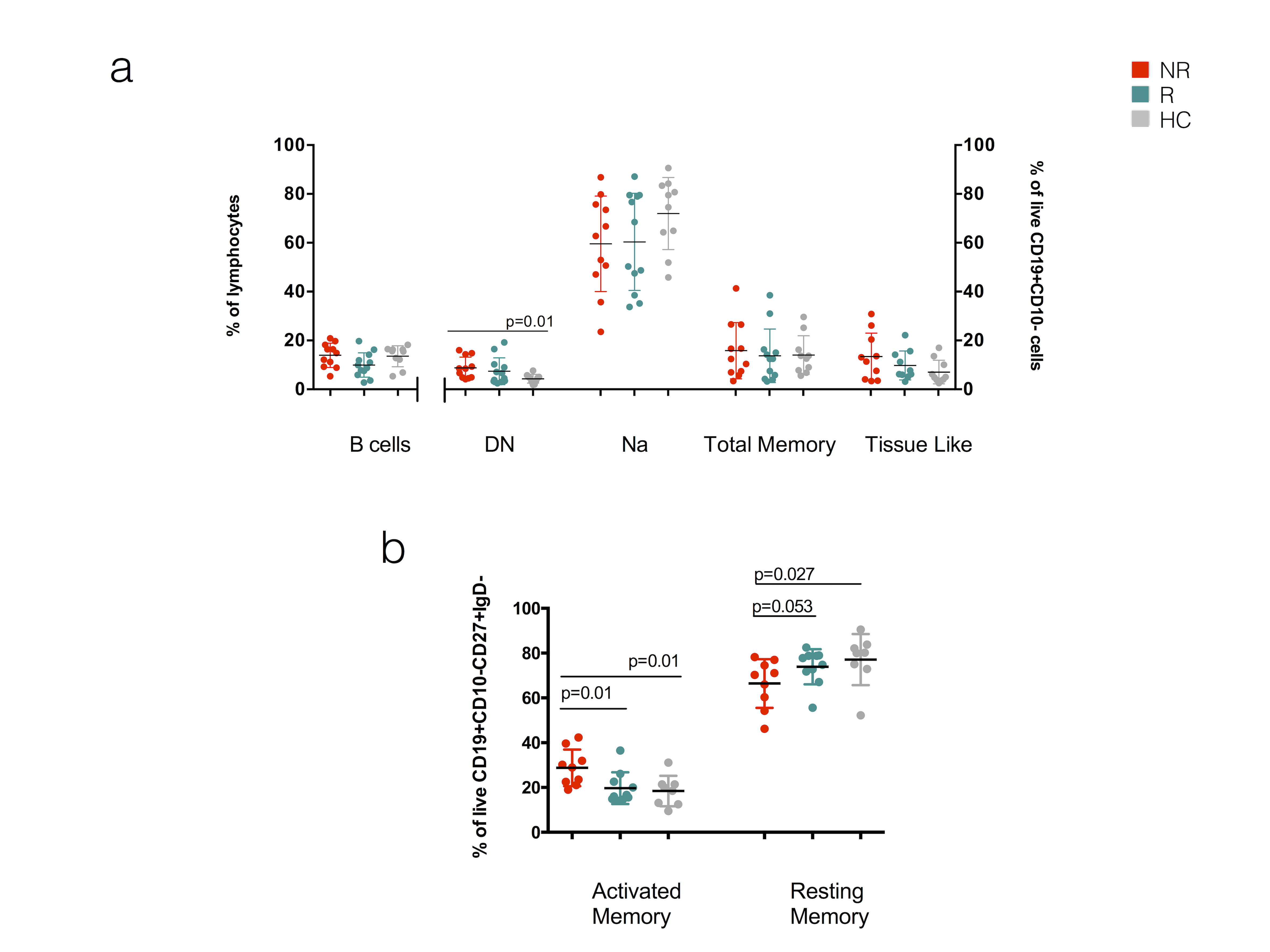

Supplement: Figure S5 — Scatter dot plot in panel (A) shows frequencies of total B cells (live, CD10−, CD20+); and among B cells: double negative (CD27−, IgD−), total naive (CD27−, IgD+), class switched CD27+ memory B cells (CD27+, IgD−), tissue like (CD27−, IgD−, CD21−). In panel (B) activated memory (CD27+, IgD−, CD21−) and resting memory (CD27+, IgD−, CD21+) (two tailed Mann–Whitney test for comparisons) are shown. [file image_5.tiff]

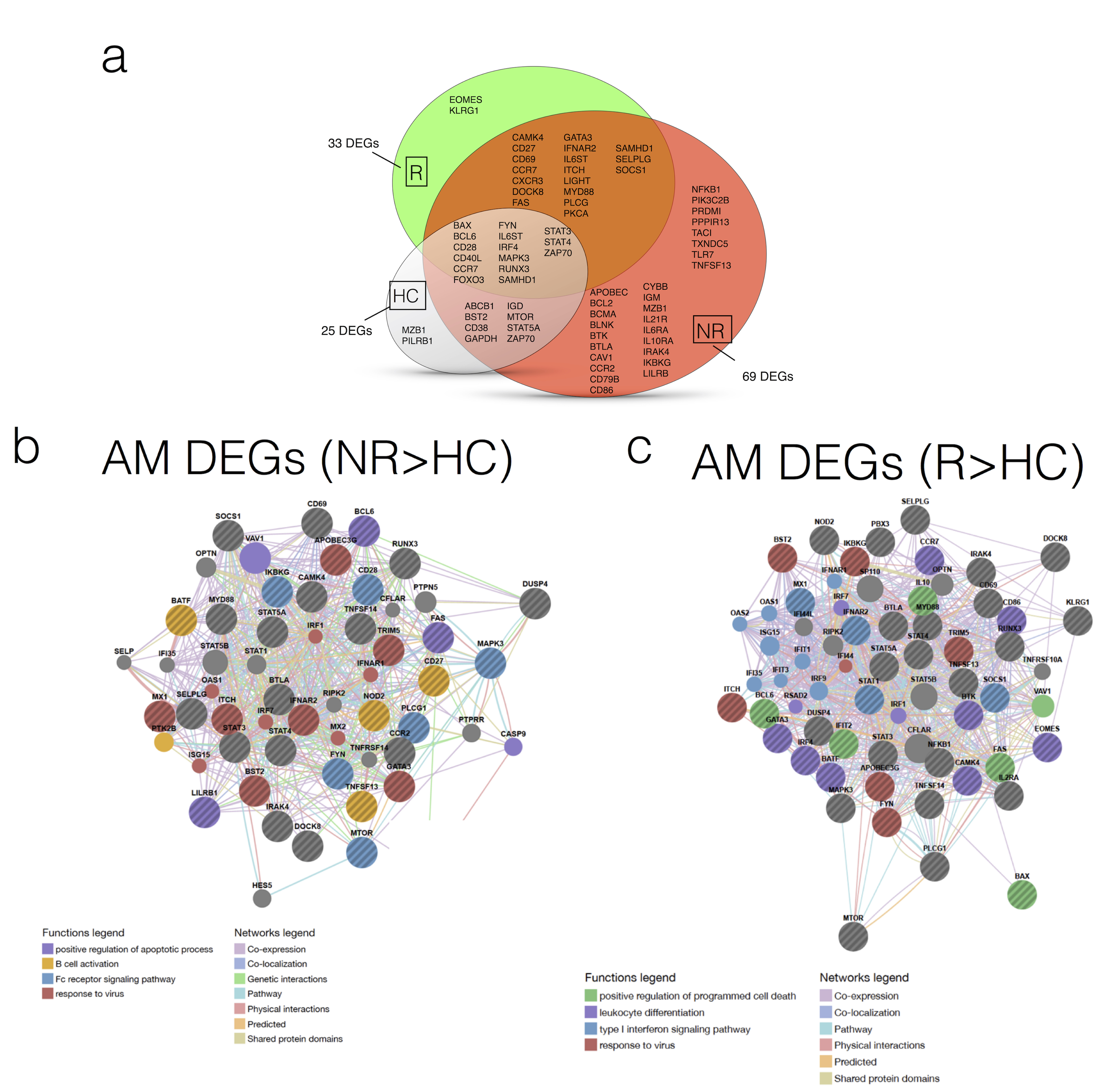

Supplement: Figure S6 — Activated memory (AM) vs. resting memory (RM) differentially expressed genes (DEGs) in healthy controls (HC), R and NR. Venn Diagram in panel (A) shows DEGs found when gene expression of AM and RM are compared between HIV R, HIV NR, and HC. Only ANOVA analysis with a p value < 0.05 are shown. (B,C) gene set enrichment analysis of DEGs derived from activated memory vs. resting memory of HIV NR vs. HC (B) and HIV R vs. HC (C) are shown. [file image_6.jpeg]

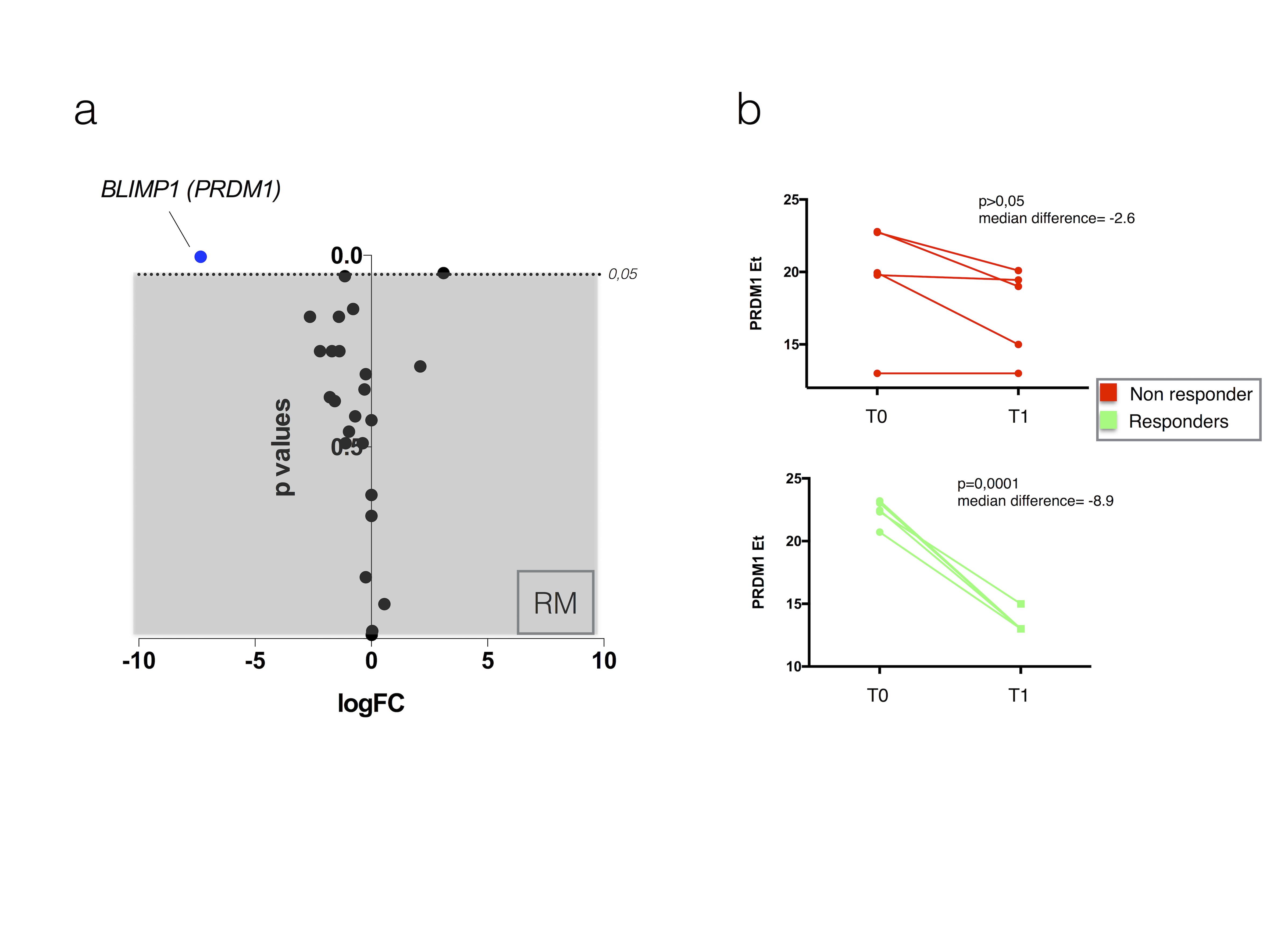

Supplement: Figure S7 — Post vaccination (T1) PRDM1 (BLIMP1) gene expression is reduced in resting memory (RM) of HIV-infected children compared to baseline (T0). In panel (A), volcano plot shows differences in gene expression between T1 and T0 in selected genes from sorted RM. In panel (B) paired analysis in R (green) and NR (red) of PRDM1 are shown. P-Values and median differences derive from Wilcoxon paired t test performed by graphpad (prism 6.0). [file image_7.tiff]

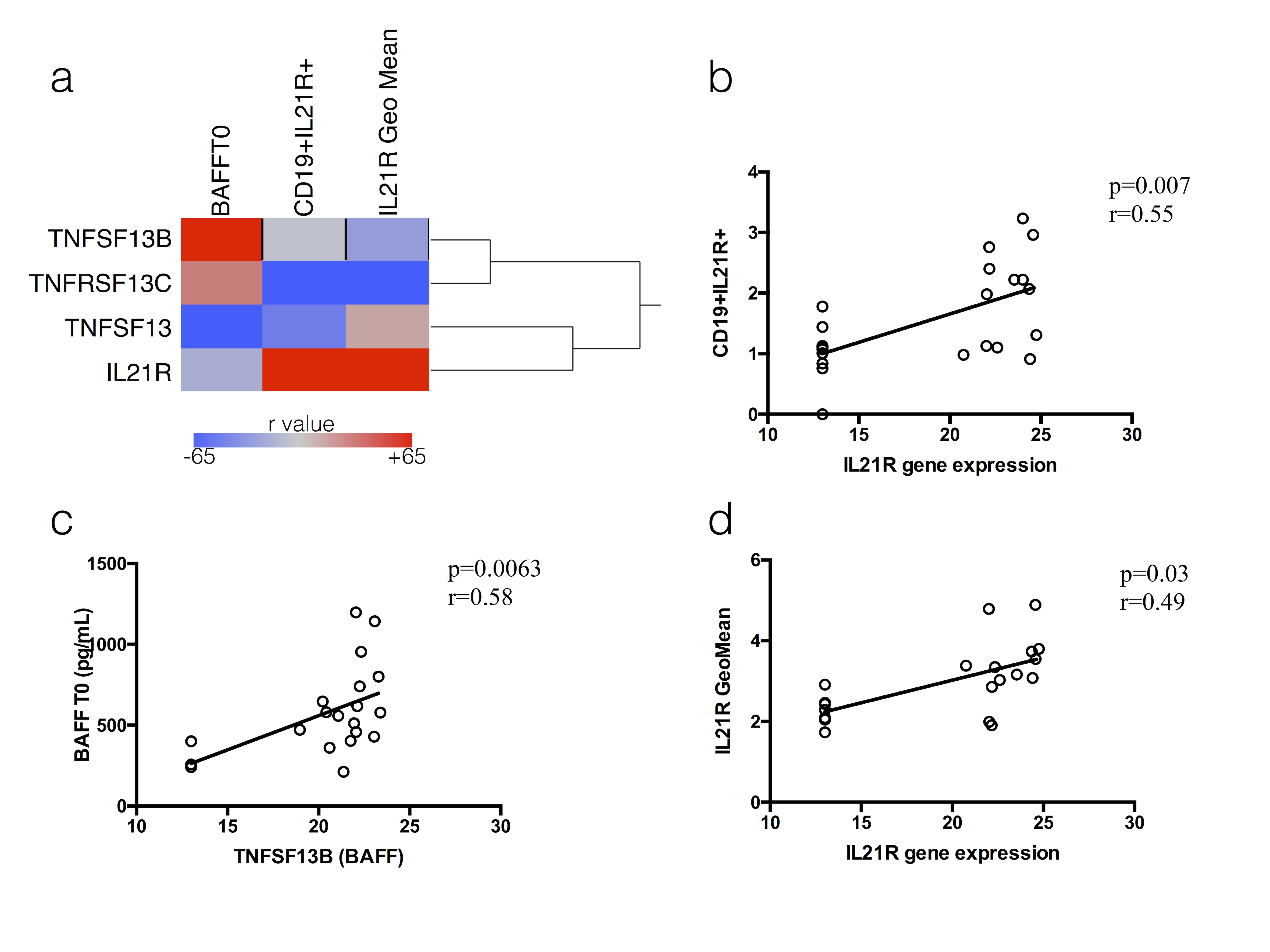

Supplement: Figure S8 — IL21R and BAFF (TNFSF13B) gene expression in resting memory (RM) correlates with surface molecules and plasma levels. In panel (A) heatmap analysis shows r values resulting from pairwise Spearman correlations between the aforementioned observations. In panels (B–D), correlation dot plots of the afore mentioned analysis are shown. In the graph gene expression resulting from RM is shown. [file image_8.tiff]
