# Supplementary material for: Perturbation of B Cell Gene Expression Persists in HIV-Infected Children Despite Effective Antiretroviral Therapy and Predicts H1N1 Response
Source: Front Immunol. 2017 Sep 11;8:1083. doi: 10.3389/fimmu.2017.01083 (PMC5600985; doi:10.3389/fimmu.2017.01083)
Supplement: Table S1 — The full list of genes, alias names and assay codes used for multiplexed principal component analysis are shown in the table. [file table_1.pdf]

GENE	ALIAS	TAQMAN ASSAY NAME	GENE	ALIAS	TAQMAN ASSAY NAME
BCL6		Hs00277037_m1	CD27		Hs00154297_m1
CD40L	CD154	Hs00163934_m1	CAMK4		Hs00174318_m1
CXCR3		Hs00171041_m1	CAV1	BSCL3	Hs00971716_m1
BCL2		Hs99999018_m1	NOD2		Hs01550762_g1
IL10		Hs00961622_m1	MZB1		Hs00414907_m1
SAMHD1		Hs00210019_m1	TXNDC5		Hs01046709_mH
TNFSF13	APRIL	Hs00182565_m1	BTK		Hs00975865_m1
CD86	B7-2	Hs01567025_m1	IKBKG	NEMO	Hs00415849_m1
ABCB1	MDR1	Hs00184500_m1	CCR7		Hs00171054_m1
CCR2		Hs00356601_m1	IFNAR2		Hs01022060_m1
CD74	DHLAG	Hs00959498_g1	BATF	BCL2L4	Hs00232390_m1
CXCL10	IP10	Hs00171042_m1	EOMES		Hs00172872_m1
LILRB1	ILT2	Hs01848117_s1	NFKB1		Hs00765730_m1
TRIM5		Hs01552559_m1	SOCS1		Hs00705164_s1
CD79B		Hs01058826_g1	TLR7		Hs00152971_m1
IL10RA		Hs00155485_m1	TLR9		Hs00152973_m1
SELPLG		Hs00380945_m1	BLNK		Hs00179459_m1
BST2	Tetherin	Hs00171632_m1	IGD	IGHD	Hs00920518_g1
CD38		Hs01120071_m1	IRAK4		Hs00211610_m1
DUSP4		Hs01027785_m1	FOXO3		Hs00921424_m1
IFIT2		Hs00533665_m1	IL2RA	CD25	Hs00166229_m1
IL6ST	gp130	Hs00174360_m1	PDL1	CD274	Hs00228839_m1
IRF4		Hs01056533_m1	PPP3CA		Hs00174223_m1
ITCH		Hs00395201_m1	TNFSF13B	BAFF	Hs00198106_m1
PRDM1	BLIMP1	Hs00153357_m1	IGM		Hs00941538_g1
PTEN		Hs02621230_s1	IRAK3		Hs00936103_m1
BTLA		Rh02889477_m1	MYD88		Hs01573837_g1
FAS		Hs00531110_m1	NKRF		Hs00213907_m1
GATA3		Hs00231122_m1	PILRB		Hs00273801_m1
IL6		Hs00985639_m1	PPPIR13B		Hs00367408_m1
IL6RA		Hs00169842_m1	TIRAP		Hs00364644_m1
STAT3		Hs01047580_m1	TNFRSF13C	BAFFR	Hs00606874_g1
STAT5A		Rh02844611_m1	CYBB		Hs00166163_m1
PAX5		Hs00277134_m1	FYN		Hs00941600_m1
CD28		Hs00174796_m1	MTOR		Hs00234508_m1
CD69		Hs00934033_m1	MX1		Hs00895608_m1
DOCK8		Hs00298892_m1	PKC A	PRKCA	Hs00925195_m1
HAVCR2	TIM3	Hs00958623_m1	TNFRSF4	OX40	Hs00533968_m1
KLRG1		Rh00929962_m1	MAPK3	ERK1	Hs00533968_m1
STAT1		Hs01013996_m1	PDCD1	PD1	Hs00169472_m1
STAT4		Rh02896026_m1	PIK3C2B	PI3K	Hs00898518_m1
BCMA	TNFRSF17	Hs00171292_m1	PLCG		Hs01008225_m1
GAPDH		Hs99999905_m1	RUNX3		Hs00231709_m1
APOBEC3G		Hs00222415_m1	SYK		Hs00374292_m1
LAG3		Hs00158563_m1	ZAP70		Hs00896345_m1
LIGHT	TNFRSF14	Hs00998604_m1	PBX3		Hs00608415_m1
TACI	TNFRSF13B	Hs00963364_m1	IL21R		Hs00222310_m1
BAX		Hs00180269_m1	RORC		Hs01076112_m1
