# Supplementary material for: Perturbation of B Cell Gene Expression Persists in HIV-Infected Children Despite Effective Antiretroviral Therapy and Predicts H1N1 Response
Source: Front Immunol. 2017 Sep 11;8:1083. doi: 10.3389/fimmu.2017.01083 (PMC5600985; doi:10.3389/fimmu.2017.01083)
Supplement: Table S2 — Differentially expressed genes (DEGs) between cell subsets in HIV, healthy controls (HC), R and NR. Table shows DEGs in all groups (HIV, HC, HIV NR, HIV R) when subsets were compared. In the table, cells are highlighted in orange when p values were <0.01 and not highlighted when p value <0.05. Outlined cells define an opposite relation compared to the one in the headline. [file table_2.pdf]

p<0.01
p<0.05
subset<subset

HC

AM>Rem	DN>Rem	Naïve>Rem	Naïve>DN	Naïve>AM	DN>AM
CD40L	IGD	IGD	no DEGs	**CD28**	**CD28**
CD28	PILRB	PILRB		IGD	**CD40L**
PILRB	CCR7	BCL2		IRAK3	IGD
FOXO3	FOXO3	FOXO3		SOCS1	IL6
CD38	PIK3C2B	CD69		TIRAP	**CAMK4**
STAT5A	RUNX3	PIK3C2B		**CAMK4**	TNFSF13
ABCB1	GAPDH	BCL6		PRDM1	
MZB1	BCL2	CCR7		CD40L	
MAPK3	STAT5A	BLNK		BTLA	
SAMHD1	BLNK	BTLA		TNFSF13	
STAT4	BCL6	STAT4		CXCL10	
BCL6	MAPK3	RUNX3		IL6	
GAPDH	MYD88	TNFSF13			
BAX	STAT4	GAPDH			
RUNX3	IL6RA	MAPK3			
IL6RA	BST2	IRAK3			
IL6ST	IRF4	CXCR3			
CCR7	BAX	TIRAP			
IRF4	BTLA	MYD88			
FYN	ABCB1	BAX			
BLNK	CD69	CD38			
BST2	TNFSF13	MX1			
STAT3	ITCH	ABCB1			
ZAP70	IL6	CD79B			
	MTOR	STAT1			
	CD86	STAT3			
	SYK	PTEN			
	MX1	IL6ST			
	CD38	SYK			
	STAT3	BST2			
	CD79B	ITCH			
	DUSP4	IL21R			
	SAMHD1	IFIT2			
	PTEN	IL10RA			
	IFIT2	PLCG			
	LILRB1	IRF4			
	NFKB1	STAT5A			
	MZB1	IL6			
	IL10RA	CD74			
	TACI	DOCK8			

HIV

AM>Rem	DN>Rem	Naïve>Rem	Naïve>DN	Naïve>AM	DN>AM
CD28	CCR7	IGD	IRAK3	CD28	CD28
CD40L	IL6ST	IL6ST	IGD	CD40L	CD40L
PKC A	MTOR	BTLA	CD79B	PKC A	PKC A
IL6ST	STAT3	CD69	TNFRSF4	CAMK4	GATA3
FYN	IGD	LILRB1	IL6RA	IRAK3	CAMK4
PLCG	MYD88	CD79B	IL10RA	IL6RA	CD27
SELPLG	IRAK4	IRAK3	IFIT2	IL6	SELPLG
GATA3	MAPK3	TNFSF13	CAMK4	IGD	FYN
IRAK4	STAT4	BLNK	CD69	SELPLG	PLCG
STAT3	FYN	STAT3	PPPIR13B	KLRG1	CCR2
CCR7	BTLA	PLCG	LILRB1	ZAP70	IL6ST
CAMK4	CAV1	MAPK3	BTLA	GATA3	PRDM1
MTOR	RUNX3	CCR7		EOMES	SAMHD1
STAT4	DUSP4	TLR7		PRDM1	EOMES
MAPK3	PLCG	IRAK4		CD27	KLRG1
LILRB1	LILRB1	FYN		CCR2	TRIM5
RUNX3	TNFSF13	PAX5			IL6
MYD88	IL6RA	ABCB1			ZAP70
CD69	SOCS1	BCL6			IL10RA
ZAP70	CD69	SYK			IFIT2
IL6RA	TLR7	MTOR			TACI
SYK	STAT5A	IL6			MZB1
TNFSF13	NFKB1	MYD88			APOBEC3 G
BLNK	IRF4	CYBB			PPPIR13B
FAS	ABCB1	LIGHT			CD38
FOXO3	LIGHT	PPPIR13B			BCMA
GAPDH	BLNK	STAT4			NOD2
LIGHT	PAX5	FOXO3			IRAK4
SAMHD1	IFNAR2	DOCK8			CXCR3
PPPIR13B	FAS	PIK3C2B			
IFNAR2	SYK	SOCS1			
APOBEC3G	BTK	PILRB			
BCL6	IL6	APOBEC3G			
BAX	ITCH	RUNX3			
CD27	SELPLG	FAS			
DUSP4	GAPDH	PTEN			
DOCK8	TNFRSF4	STAT5A			
STAT5A	IGM	MX1			
IRF4	BCL6	ITCH			
PAX5	FOXO3	BTK			
TACI	BAX	IRF4			
MX1	CYBB	IL10RA			
BTLA	CD86	BAX			
TLR7	BST2	GAPDH			
ITCH	ZAP70	BST2			
ABCB1	PIK3C2B	CAV1			
CAV1	PDCD1	IFIT2			
PRDM1	PKC A	BCL2			
CYBB	DOCK8	STAT1			
SOCS1	PILRB	NFKB1			
CD79B	PPPIR13B	TACI			
IGD	BATF	CD38			
MZB1	CD79B	IFNAR2			
NFKB1	APOBEC3G	CD74			
BST2	MX1	GATA3			
BTK	TXNDC5	SELPLG			
CD38	PTEN	DUSP4			
CD86		TRIM5			
BCMA		IL21R			
IGM		TXNDC5			
PIK3C2B		MZB1			
EOMES		CXCR3			
KLRG1		IKBKG			
IL10RA		BATF			
TXNDC5		IGM			
CXCR3		TNFRSF13C			
TRIM5					
CCR2					
PILRB					
IFIT2					
STAT1					
BATF					
IKBKG					
PPP3CA					
NOD2					
IL21R					
IL10					
HAVCR2					

p<0.01
p<0.05
subset<subset

NR

AM>Rem	DN>Rem	Naïve>Rem	Naïve>DN	Naïve>AM	DN>AM
CD28	MTOR	IGD	IRAK3	CD28	CD28
IL6ST	IGD	IL6ST	CD79B	IL6	PLCG
FYN	IL6ST	IRAK3	IGD	IRAK3	GATA3
PLCG	CCR7	CD69	BLNK	CD40L	SELPLG
GATA3	TNFSF13	BTLA	TRIM5	IGD	CD40L
MTOR	IL6RA	BLNK	PPPIR13B	CAMK4	PKCA
SELPLG	CD69	LILRB1	TNFRSF4	PKCA	CD27
IGD	MYD88	TNFSF13	PIK3C2B	GATA3	IL6
CD69	FYN	MTOR	IL10RA	EOMES	FYN
TNFSF13	TNFRSF4	CD79B	BTLA	ZAP70	TRIM5
APOBEC3G	STAT5A	PLCG	PLCG	SELPLG	CCR2
STAT3	NFKB1	CCR7	BCL6		PPPIR13B
PKCA	LILRB1	BCL6	LILRB1		CAMK4
LILRB1	BTLA	SYK	CD69		IL10RA
IRAK4	CAV1	FYN	PTEN		IL6ST
CCR7	RUNX3	IL6	FOXO3		BCMA
STAT4	STAT3	MYD88	PAX5		TNFRSF4
BCL6	IL6	MAPK3	IFIT2		CXCR3
MAPK3	MAPK3	APOBEC3G	SYK		
SYK	STAT4	STAT3			
PPPIR13B	SYK	PPPIR13B			
MYD88	BST2	ABCB1			
BLNK	APOBEC3G	TLR7			
CD40L	GATA3	CYBB			
RUNX3	TLR7	PIK3C2B			
BTLA	IRAK4	IRAK4			
CAV1	PLCG	STAT4			
IL6RA	BCL6	LIGHT			
CAMK4	ABCB1	PAX5			
STAT5A	CYBB	STAT5A			
BST2	BLNK	RUNX3			
LIGHT		BCL2			
GAPDH		DOCK8			
IL10RA		BST2			
TLR7		PTEN			
NFKB1		IL10RA			
ZAP70		ITCH			
TACI		FOXO3			
ABCB1		GAPDH			
ITCH		MX1			
DOCK8		NFKB1			
SAMHD1		BTK			
BAX CYBB		TACI			
CD27 BTK		PILRB			
IFNAR2		CD38			
CD79B		IRF4			
MX1		TXNDC5			
IGM		CAV1			
MZB1		IFIT2			
BCMA		BAX			
PIK3C2B		GATA3			
PRDM1		SOCS1			
PAX5		SELPLG			
DUSP4		FAS			
FOXO3		MZB1			
IRF4		STAT1			
BCL2		IFNAR2			
SOCS1		TRIM5			
CD38		DUSP4			
CXCR3		IL21R			
CD86					
FAS					
IKBKG					
CCR2					
TRIM5					
TXNDC5					
IL21R					

R

AM>Rem	DN>Rem	Naïve>Rem	Naïve>DN	Naïve>AM	DN>AM
CD28	IRF4	IGD	IL6RA	CAMK4	CD28
STAT4	SOCS1	IL6RA	DUSP4	CD28	CD40L
PKCA	NFKB1	CD69	CD86	PKCA	PKCA
SELPLG	STAT4	CD86	CAMK4	CD27	CD27
IL6ST	CAV1		PDCD1	IL6RA	GATA3
ZAP70	IGD		NFKB1	KLRG1	CAMK4
CD40L	IL6ST		BATF	CD40L	KLRG1
CAMK4	CCR7			SELPLG	SELPLG
IRF4	STAT3			CD86	ZAP70
KLRG1				IGD	IGD
CD69				STAT4	IFIT2
IRAK4				ZAP70	EOMES
FYN				DUSP4	SAMHD1
GATA3				RUNX3	
MAPK3				SAMHD1	
IFNAR2				FYN	
RUNX3				MTOR	
PLCG				PRDM1	
BCL6				BATF	
LIGHT				NOD2	
FOXO3				ITCH	
ITCH				IRF4	
STAT3					
DOCK8					
BAX					
EOMES					
CD27					
SOCS1					
FAS					
MYD88					
CXCR3					
CCR7					
SAMHD1					
