# Supplementary material for: Perturbation of B Cell Gene Expression Persists in HIV-Infected Children Despite Effective Antiretroviral Therapy and Predicts H1N1 Response
Source: Front Immunol. 2017 Sep 11;8:1083. doi: 10.3389/fimmu.2017.01083 (PMC5600985; doi:10.3389/fimmu.2017.01083)
Supplement: Table S3 — Differentially expressed genes between study groups. Note: genemania.org gene set enrichment analysis (GSEA) In every graph presented throughout the paper, circles with diagonal lines define genes included in the GSEA. Its function is described legend below the graph. The solid circles represent genes resulted to be involved in the pathways. Networks among the genes are defined by colored lines, further explained by legends. [file table_3.pdf]

AM

NR>HC	R>HC	R>NR
TNFSF13	IL2RA	IL2RA
MX1	STAT4	CCR2
SELPLG	NOD2	PPP3CA
IRAK4	SELPLG	
CCR2	TNFSF13	
STAT4	CD69	
FYN	IFNAR2	
MYD88	MYD88	
NOD2	BCL6	
BCL6	DOCK8	
STAT3	MX1	
ITCH	IRAK4	
APOBEC3G	IKBKG	
STAT5A	RUNX3	
FAS	TRIM5	
MAPK3	SOCS1	
RUNX3	IFIT2	
TRIM5	FYN	
IKBKG	BATF	
DOCK8	ITCH	
BATF	CAMK4	
BST2	PLCG	
PLCG	KLRG1	
CD69	MAPK3	
GATA3	CD86	
SOCS1	NFKB1	
CAMK4	STAT5A	
BAX	DUSP4	
BTLA	BTK	
MTOR	LIGHT	
CD27	GATA3	
LIGHT	MTOR	
DUSP4	IRF4	
CD28	FAS	
IFNAR2	APOBEC3G	
LILRB1	BST2	
	CCR7	
	STAT3	
	BAX	
	STAT1	
	IL10	
	BTLA	
	PBX3	
	EOMES	

Rem

NR>HC	R > HC	R>NR
-	BCL2	CD69
	PILRB	IGD
	IL6RA	BCL6
	CD86	BCL2
	ABCB1	BTLA
	CCR7	ABCB1
	PIK3C2B	IL2RA
	IGD	BTK
	IL2RA	MTOR
	BCL6	IL6RA
	STAT5A	CCR7
	IFIT2	ITCH
	BLNK	TNFSF13
		MX1
		LIGHT
		RUNX3
		FYN
		PIK3C2B
		APOBEC3G
		STAT4
		PRDM1
		MYD88
		CD86
		IFNAR2
		BST2

DN

NR>HC	R>HC	R>NR
PIK3C2B	IL2RA	IL2RA
HAVCR2	IFNAR2	TRIM5
		PIK3C2B

Naïve

NR>HC	R>HC	R>NR
		SYK
		ITCH
		BTLA
		IL10RA

PBMCs

NR>HC	R>HC	R>NR
	IL2RA	BCMA
	CCR7	IL2RA
		IFNAR2

Total B

NR>HC	R>HC	R>NR
SELPLG	IL2RA	IL2RA
	KLRG1	IL6
